# Single-cell lineage tracing in tumor organoids identifies expansion states depicting the origin and progression of lung adenocarcinoma

**DOI:** 10.3389/fimmu.2026.1903250

**Published:** 2026-09-07

**Authors:** Wutao Chen, Peng Li, Hejian Zhang, Yumeng Sheng, Guoliang Yang, Jing Hu, Bin Yu

**Affiliations:** 1 State Key Laboratory of Systems Medicine for Cancer, Shanghai Cancer Institute, Renji Hospital, School of Medicine, Shanghai Jiao Tong University, Shanghai, China; 2 Department of Urology, Renji Hospital, Shanghai Jiao Tong University School of Medicine, Shanghai, China; 3 Department of Anesthesiology, Renji Hospital, Shanghai Jiao Tong University School of Medicine, Shanghai, China; 4 Shanghai Key Laboratory for Cancer Systems Regulation and Clinical Translation (CSRCT), Shanghai, China

**Keywords:** lineage tracing, lung adenocarcinoma, organoids, single-cell RNA sequencing, tumor initiation

## Abstract

**Introduction:**

Only a subset of epithelial cells can initiate progressive lung adenocarcinoma (LUAD), yet the founder cell states that confer this competence remain poorly defined.

**Methods:**

We established a barcoded *Kras*^G12D^, *Trp53* loss mouse LUAD organoid model that couples 15-nucleotide lineage tracing with single-cell RNA sequencing (scRNA-seq) before and after syngeneic transplantation. Candidate markers for expansion states were further evaluated using wild-type lung organoid, spatial transcriptomics, and independent human scRNA-seq datasets.

**Results:**

Barcoded organoids generated LUAD-like tumors. Paired barcode recovery identified expanded, diminished and failed lineages and showed that expanded lineages were enriched for surfaceome genes associated with stemness. Among candidate surface markers, *Plxna2* showed the strongest enrichment in baseline organoid cells that expanded after transplantation and marked cells with stronger stemness features. These cells underwent stage-specific reprogramming, with early EMT and inflammatory adaptation followed by late metabolic and MYC programs rebound within an immunosuppressive microenvironment. Wild-type lung organoid analysis showed that *Plxna2* marks an alveolar remodeling state sharing expansion signature. Spatial transcriptomics placed *Plxna2*^high^ tumor epithelial cells in EMT-rich and stromal-interface niches. Human LUAD scRNA-seq cohorts further showed enrichment of *PLXNA2*^high^ malignant cells within expansion and stemness states.

**Discussion:**

These findings identify *PLXNA2* as a candidate marker of expansion state and provide a lineage-based framework for discovering tumor-initiating programs in LUAD.

## Introduction

Lung cancer remains the leading cause of cancer death worldwide, with approximately 2.5 million new cases and 1.8 million deaths estimated in 2022 (1). Lung adenocarcinoma (LUAD) is now the dominant histological subtype in many populations (2). Current guidelines integrate surgery, radiotherapy, platinum-based chemotherapy, immune-checkpoint blockade, and an expanding set of genotype-matched agents for EGFR, ALK or KRAS alterations (3–6). Yet these therapeutic gains have not eliminated relapse or incomplete responses. A central biological problem therefore remains unresolved: why do only particular epithelial cells or subclones convert oncogenic injury into durable tumor growth, while many apparently transformed cells fail to establish progressive disease? Addressing this question requires preclinical systems that resolve fate at single-cell and clonal resolution.

Genetically engineered mouse models (GEMMs) have provided an essential foundation for this work. Conditional activation of oncogenic Kras and compound Kras activation with Trp53 loss recapitulate key features of LUAD initiation, progression and metastatic competence (7, 8). However, autochthonous GEMMs are slow and resource-intensive. Lung organoid platforms have helped bridge this gap by enabling epithelial cells from normal or engineered lungs to be cultured, perturbed, transplanted and molecularly profiled under controlled conditions. Recent organoid studies have shown that KRAS activation can induce LUAD transcriptional programs in lung epithelial progenitors (9, 10). Even so, organoids and transplant models usually describe which states are present, not which founder states have tumor-initiating potential. A method that connects pre-existing organoid states to eventual tumor fate is needed to turn these platforms from descriptive models into models of tumor initiation.

Lineage tracing approaches can help fill this gap. Modern high-complexity barcoding and single-cell readouts now allow clonal identity and transcriptional state to be measured together (11, 12). In cancer, barcoding and multi-omic lineage tracing have begun to uncover how rare clones expand or adapt to therapy (13–15). The 15-nucleotide (15N) barcode strategy used here is complementary to these approaches. By introducing barcodes into KRAS/TP53-mutant lung organoids before transplantation, this system can compare the baseline states of lineages that later expand, diminish or fail. As a result, tumor fate can be traced back to founder organoid cells before *in vivo* selection has erased the initiating state.

Here we establish a barcoded *Kras*^G12D^, *Trp53* loss LUAD organoid model that couples 15N lineage tracing with single-cell transcriptomics across baseline organoids and tumor engraftments. We classify lineages by tumor recovery and use fate-defined contrasts to nominate *Plxna2*^high^ cells as a candidate marker for expansion state. We further show that Plxna2 marks an alveolar remodeling compartment during wild-type organoid formation, maps in tumors to EMT-like epithelial cells positioned near stromal niches, and is enriched in expansion state malignant cells in human LUAD cohorts. These results provide a preclinical framework for LUAD initiation and suggest that tumor-initiating competence may arise from an epithelial state that precedes tumor growth and is later reshaped by the tumor microenvironment (TME).

## Results

### Establishment of a barcoded mouse lung adenocarcinoma organoid model

**Figure 1:**
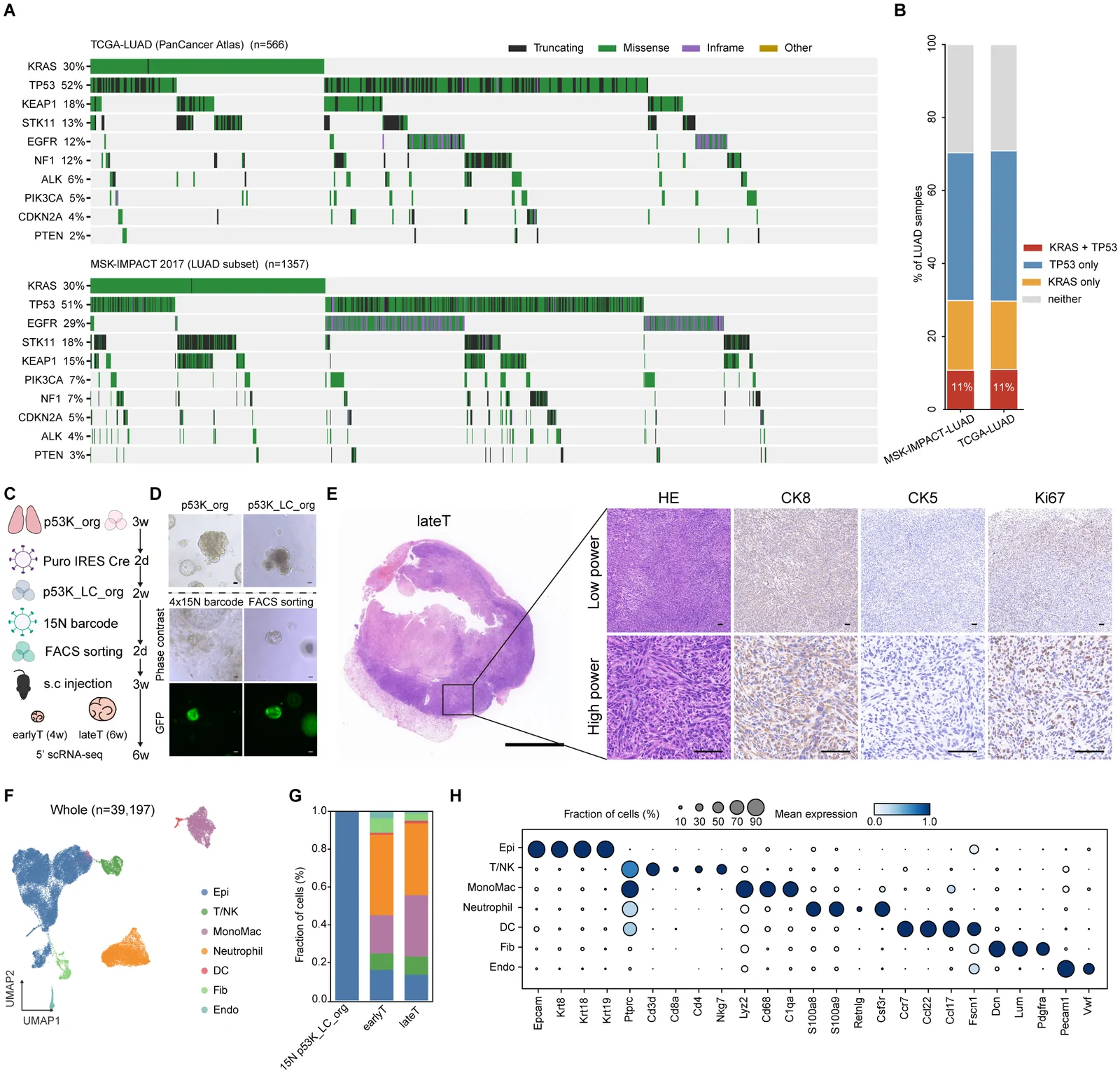
Establishing a barcoded KRAS/TP53 lung adenocarcinoma organoid model. **(A)**, Oncoprints of recurrent LUAD driver alterations in TCGA-LUAD (16) and the MSK-IMPACT LUAD subset (17). **(B)**, Proportion of LUAD cases carrying KRAS and/or TP53 alterations in the two cohorts. **(C)**, Experimental workflow and timeline for generating barcoded p53K_LC organoids and profiling transplanted tumors. Lung-derived p53K_org organoids from *Trp53*^fl/fl^, *Kras*^LSL-G12D^ mice (n = 3 donor mice) were cultured for 3 weeks before Puro-IRES-Cre transduction. After an additional 2 weeks, the resulting p53K_LC_org organoids were transduced with the 15N barcode library. EGFP-positive barcoded cells were enriched by FACS, cultured in organoid culture for 3 weeks, and transplanted subcutaneously into syngeneic mice (n = 6 recipients). Early- and late-stage tumors were collected at 4 and 6 weeks after transplantation, respectively (n = 3 tumors per time point), for 5′ scRNA-seq and targeted 15N barcode sequencing. **(D)**, Representative phase-contrast and GFP images during organoid culture, lentiviral infection and FACS sorting. Scale bar, 50 μm. Organoid line was established from 3 independent donor mice. **(E)**, Representative histology and immunohistochemistry images of a late-stage tumor, showing CK8, CK5 and Ki67 staining at low and high magnification. Scale bar, 50 μm. **(F)**, UMAP of the whole single-cell atlas colored by major cell class. **(G)**, Major cell-class composition across 15N p53K_LC_org, early-stage tumor and late-stage tumor samples. **(H)**, Dot plot of canonical marker genes used to annotate epithelial and tumor microenvironment compartments. .

KRAS activation and TP53 loss are among the most clinically relevant genetic events in LUAD and have been widely modeled using conditional *Kras*^G12D^ activation combined with *Trp53* deletion (7, 8). Organoid platforms have further shown that alveolar type 2 (AT2) cells from genetically engineered mouse models (GEMMs) can generate LUAD-like tumors after transplantation (9, 10). Analysis of the TCGA-LUAD and MSK-IMPACT LUAD cohorts showed that KRAS and TP53 alterations co-occurred in 11% of LUAD cases (16, 17), supporting the clinical relevance of this genetic backbone (**Figures 1A, B**).

To build a tractable founder organoid model for fate mapping, we established lung organoids from *Trp53*^fl/fl^; *Kras^LSL-G12D/+^* mice, hereafter p53K_org. The experimental workflow is summarized in **Figures 1C, D**. p53K_org were infected with Puro-IRES-Cre to generate p53K_LC_org carrying *Trp53* deletion and *Kras*^G12D^ activation (**Supplementary Figures 1A–C**). To track the fate of individual organoid cells after transplantation, we then infected p53K_LC_org at MOI = 1 with a 15N barcode reporter composed of a miniP promoter, a short spacer, a 15N barcode and an EGFP reporter (**Supplementary Figure 1D**). Because the barcode sequence was positioned immediately downstream of the miniP promoter, paired 5’ scRNA-seq and targeted barcode sequencing enabled recovery of transcriptomes and lineage barcodes from the same cells (**Supplementary Figure 1E**).

GFP-positive barcoded p53K_LC_org cells were enriched by flow cytometry and injected subcutaneously into syngeneic C57BL/6 mice to generate tumors (**Figure 1E**; **Supplementary Figure 1F**). We sampled tumors at early formation and at a late stage based on tumor size. The late-stage tumors showed malignant histologic features, including transformed nuclear morphology, prevalent CK8 expression, minimal CK5 expression, and high Ki67 staining, in keeping with proliferative LUAD-like growth (18, 19) (**Figure 1E**).

We profiled the 15N p53K_LC_org, early-stage tumor (earlyT) and late-stage tumor (lateT) for 5’ single-cell RNA sequencing (scRNA-seq) and paired targeted lineage 15N barcode sequencing. We obtained a total of 39,197 cells across the profiled samples, including epithelial cells and tumor-microenvironment compartments (**Figures 1F–H**). We annotated cells using canonical markers including *Epcam*, *Krt8* (epithelial cells), *Cd3d*, *Cd8a*, *Nkg7* (T and NK cells), *Lyz2*, *Cd68*, *C1qa* (monocytes and macrophages), *S100a8*, *S100a9*, *Retnlg* (neutrophils), *Ccr7*, *Ccl22* (regulatory dendritic cells), *Dcn*, *Lum*, *Pdgfra* (fibroblasts), *Pecam1* and *Vwf* (endothelial cells). Epithelial cells displayed higher CNV burdens compared to the host TME reference, indicating genomic distinction from host-derived epithelial cells (**Supplementary Figures 1G, H**).

### scRNA-seq reveals epithelial heterogeneity during organoid-to-tumor progression

**Figure 2:**
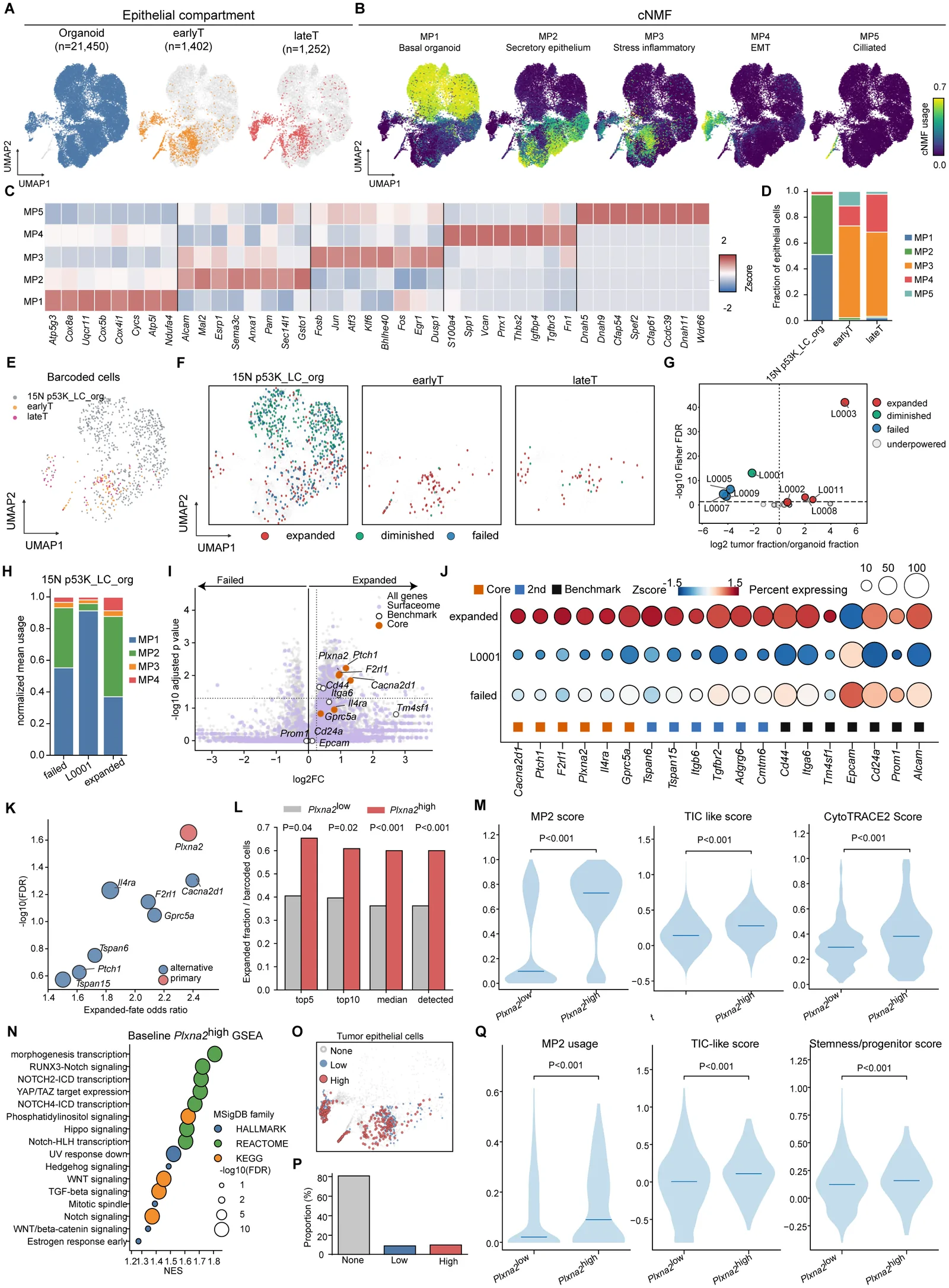
Single-cell lineage tracing identifies expansion-primed epithelial states and nominates Plxna2 as a surface marker candidate. **(A)**, UMAP of epithelial cells across organoid, early tumor and late tumor stages. **(B)**, UMAP projection of five meta-programs (MPs), annotated as MP1 basal organoid, MP2 secretory epithelium, MP3 stress-inflammatory, MP4 EMT and MP5 ciliated programs. **(C)**, Heat map of representative genes defining the five MPs. **(D)**, Fraction of epithelial cells assigned to each dominant MP across stages. **(E)**, UMAP of barcode-detected epithelial cells across organoid and tumor stages. **(F)**, UMAPs showing barcode lineage fate assignments in organoid, early tumor and late tumor cells. **(G)**, Lineage-level fate classification using tumor recovery and enrichment statistics. **(H)**, Mean MP usage in baseline organoid cells from failed, L0001 and expanded lineages. **(I)**, Differential expression between expanded and failed baseline organoid lineage cells. Surfaceome, benchmark and core candidate markers are highlighted. **(J)**, Expression of candidate and benchmark surface markers across expanded, L0001 and failed baseline organoid cells. **(K)**, Marker prioritization based on expanded-fate enrichment and statistical support. **(L)**, Expanded-lineage enrichment among *Plxna2*^high^ versus *Plxna2*^low^ barcoded cells across alternative Plxna2 thresholds. **(M)**, MP2, TIC-like and CytoTRACE2 scores in baseline organoid *Plxna2*^high^ and *Plxna2*^low^ cells. **(N)**, GSEA of baseline *Plxna2*^high^ organoid cells, highlighting developmental, Notch/WNT, PI3K and morphogenesis-associated programs. **(O)**, UMAP of tumor epithelial cells showing *Plxna2* expression classes. **(P)**, Proportion of tumor epithelial cells with no, low but detected or high *Plxna2* expression. **(Q)**, MP2 usage, TIC-like score and stemness/progenitor score in tumor epithelial *Plxna2*^high^ and *Plxna2*^low^ cells.

We next extracted the epithelial compartment from the integrated dataset, including barcoded p53K_LC organoids, early-stage tumors and late-stage tumors (**Figure 2A**). Because tumor epithelial states often vary along continuous transcriptional axes that can be compressed by discrete graph-based clustering, we used consensus non-negative matrix factorization (cNMF) to infer recurrent gene-expression programs at single-cell resolution (20).

cNMF identified five meta-programs (MPs) with distinct transcriptional features (**Figures 2B, C**; **Supplementary Figures 2A, B**). MP1 was defined by canonical mitochondrial respiratory-chain and oxidative phosphorylation genes, including *Atp5g3*, *Cox8a*, *Uqcr11*, *Cox5b*, *Cox4i1*, *Cycs*, *Atp5l* and *Ndufa4*, matching mitochondrial pathway annotations (21). MP2 combined epithelial secretory and epithelial-identity features, with representative genes including *Mal2* (22), *Pam* (23), *Alcam* and *Esrp1* (24). MP3 was dominated by immediate-early and adaptive stress-response genes, including *Fosb*, *Jun*, *Atf3*, *Klf6*, *Bhlhe40*, *Fos*, *Egr1* and *Dusp1*, concordant with IFN-γ response, hypoxia and TGF-β pathway enrichment (25, 26). MP4 contained extracellular matrix-remodeling and EMT-associated genes, including *S100a4*, *Spp1*, *Vcan*, *Thbs2*, *Tgfbr3* and *Fn1*, in line with TGF-β-associated epithelial plasticity (27). MP5 was marked by motile-cilia and axonemal genes, including *Dnah5*, *Dnah9*, and *Wdr66*, supporting its annotation as residual normal ciliated cells or a ciliated-like epithelial state (28). MP5 cells were therefore excluded from downstream tumor-focused analyses.

The composition of these MPs supported their biological annotation. Baseline p53K_LC organoids were enriched for MP1 and MP2, whereas tumor epithelial cells displayed increased MP3 and MP4 usage (**Figure 2D**). These shifts mirror canonical cancer hallmarks, including deregulated metabolism, tumor-promoting inflammation and invasion-associated plasticity (29). To determine whether the same programs reflected patient tumor features, we projected them onto human lung cancer scRNA-seq cohorts GSE131907 (30) and GSE127465 (31). Using both module-score projection and Scissor-based phenotype transfer (32), we found that MP3 and MP4 were preferentially associated with malignant epithelial cells in patient tumors, whereas MP1 and MP2 were also detectable within malignant compartments (**Supplementary Figures 2C–F**). In contrast, MP5 mapped primarily to normal ciliated epithelial cells. We compared the epithelial cells MP with two autochthonous KP model (33, 34). The enrichment analysis showed that the epithelial programs identified in our model were also represented across epithelial states in both autochthonous KP datasets (**Supplementary Figures 2G, H**).

We then used CytoTRACE2 to evaluate relative developmental potential across tumor epithelial programs (35). MP4 showed the highest overall CytoTRACE2 score, in keeping with evidence that EMT can be coupled to cancer stem cell phenotypes (36, 37). Notably, MP2 contained the largest fraction of cells within the top 5% CytoTRACE2 scores (**Supplementary Figures 2I–L**). These results indicate that p53K_LC organoids carrying Trp53 loss and *Kras*^G12D^ activation underwent malignant transformation and acquired distinct features after transplantation.

### Paired 15N barcode sequencing and 5’ scRNA-seq unveiled tumor lineages with tumor-initiating ability

Having defined epithelial meta-programs, we next linked transcriptional states to clonal fate by pairing 5’ scRNA-seq with targeted 15N barcode sequencing. Lineage-coupled single-cell or spatial transcriptomic approaches have been used to resolve rare expanding subclones and state transitions in Kras/Trp53-driven lung tumor evolution (13, 14). From the 15N barcode library, we recovered 20 lineages comprising 1,092 barcoded epithelial cells after quality control (**Figure 2E**; **Supplementary Figure 3A**). The largest lineage, L0001, contained more than 300 cells and was detected in organoids, early-stage tumors and late-stage tumors (**Supplementary Figure 3B**). We classified lineages as expanded, diminished, failed or underpowered according to tumor engraftment and competitive recovery relative to their baseline organoid abundance (see Methods; **Figures 2F, G**). L0003, L0002, L0011 and L0008 were classified as expanded lineages, whereas L0005 and L0009 were among the failed lineages. L0001 was detected after engraftment but did not expand competitively in tumors.

Because the same barcodes were observed at baseline and after tumor growth, this dataset allowed us to ask whether fate-biased founder states were already present in organoids. In baseline organoid cells, expanded lineages contained higher proportions of MP2 and MP4 than failed lineages, whereas L0001 was dominated by the MP1 organoid-metabolic program (**Figure 2H**). We therefore compared baseline organoid cells from expanded and failed lineages to identify transcriptional features associated with tumor initiation and expansion (**Figure 2I**). To prioritize markers that could be tested prospectively, we focused on surfaceome genes, because cell-surface proteins are compatible with antibody-based isolation, flow cytometry and therapeutic target nomination (38). The comparison recovered several benchmark lung cancer stem-cell-associated surface genes, including Cd44, Itga6 and Alcam (39), indicating that lineage-defined contrasts can nominate candidate markers.

To nominate surface candidates related with the expansion state, we ranked genes by their enrichment in expanded lineage cells and by their specificity relative to non-expanded lineages. This workflow identified candidates that were more highly expressed in expanded organoids than in L0001 and failed lineages, a pattern shared by benchmark markers (**Figure 2J**; **Supplementary Figure 4A**). We then tested whether marker expression could distinguish baseline organoid cells with expanded versus failed fate. Using top 10% expression as a threshold, most candidate markers were enriched among the expanded lineage, with *Plxna2* showing the strongest combination of effect size and statistical support (OR = 2.37, FDR = 0.022; **Figure 2K**). PLXNA2 encodes a plexin-A semaphorin receptor. Plexin/semaphorin signaling has been implicated in cancer cell proliferation, migration and tumor-niche interactions (40, 41). Alternative *Plxna2* cutoffs yielded a similar pattern, with *Plxna2*^high^ epithelial cells enriched for expanded fate (**Figure 2L**). Spearman correlation analysis further showed that the candidate markers were positively correlated with one another, forming a surfaceome-associated hub that included *Cd164*, *Tgfbr2*, *Cd44*, *Itga6* and *Alcam* (**Supplementary Figures 4B, C**). These genes were also correlated with MP2 and MP4 usage (**Supplementary Figure 4D**), linking the candidate marker set to stemness programs.

We next tested whether *Plxna2*^high^ baseline cells showed a broader expansion-primed phenotype. Across the full baseline organoid epithelium, *Plxna2*^high^ cells had higher MP2 usage, TIC-like scores and CytoTRACE2 scores (**Figure 2M**). GSEA of baseline *Plxna2*^high^ cells showed enrichment of Notch, Hippo/YAP-TAZ, WNT and TGF-β-related programs (**Figure 2N**), pathways frequently linked to CSC maintenance, epithelial plasticity and therapy-resistant states (42). *Plxna2* was expressed in a relatively small fraction of tumor epithelial cells (**Figures 2O, P**). *Plxna2*^high^ tumor cells showed higher MP2 usage, TIC-like scores and stemness/progenitor scores than *Plxna2*^low^ cells (**Figure 2Q**). Thus, paired lineage and transcriptome analyses support *Plxna2*^high^ as a candidate baseline expansion state.

### Transcriptomic reprogramming of expanded lineages and immunosuppressive microenvironment remodeling

Having identified *Plxna2* as a candidate expansion marker at baseline, we next examined how expanded lineages transcriptionally transitioned from organoids to tumors. We compared tumor-derived cells with their matched baseline organoid counterparts in these lineages. Across all four lineages, tumor cells showed increased MP4 usage together with higher TGF-β, JAK-STAT and MAPK pathway scores (**Supplementary Figure 5A**). Tumor cells were also enriched for ECM remodeling, collagen assembly, PDGF signaling, TNFα/NF-κB, EMT, TGF-β and interferon-response programs, accompanied by depletion of ribosomal, translational, oxidative phosphorylation and MYC-associated programs (**Supplementary Figure 5B**). This pattern suggests an early *in vivo* adaptation state characterized by matrix remodeling, inflammatory signaling and epithelial plasticity rather than immediate biosynthetic outgrowth. This pattern parallels established links between epithelial plasticity, stromal remodeling and tumor progression (43, 44). Persistent interferon signaling has also been linked to adaptive immune resistance programs in tumors (45).

Within expanded lineages, late-stage tumor cells showed elevated stemness scores and a rebound of metabolic, translational and MYC-associated activity compared with early-stage cells (**Supplementary Figures 5C, D**). Thus, tumor expansion in our model appeared to follow a two-step transition. The early adaptation phase was marked by inflammatory, ECM-remodeling and EMT/TGF-β programs, followed by a late biosynthetic rebound with renewed translation and MYC activity (**Supplementary Figure 5E**). This late rebound fits with the role of MYC as a central regulator of cancer cell growth, biosynthetic capacity and tumor-immune interactions (46). L0001 provided a non-expanded comparator. Similar to tumor cells in the expanded lineages, L0001 tumor cells acquired interferon, EMT, TGF-β and Hedgehog programs (**Supplementary Figures 5E–H**). However, ribosomal, mitochondrial, DNA-replication and MYC programs remained depleted in L0001, indicating that acquisition of an adaptation state alone was not sufficient for competitive clonal expansion.

We next examined whether clonal expansion occurred in a remodeled immune microenvironment. Compared with normal lung, tumors showed a lower fraction of CD45+ immune cells (**Supplementary Figure 6**). Effector CD8^+^ T cells were reduced, whereas exhausted PD-1^+^ CD8^+^ T cells and immunosuppressive myeloid populations were increased. This pattern aligns with current models in which CD8^+^ T cell dysfunction and suppressive myeloid compartments limit antitumor immunity and shape therapeutic response (47, 48). Thus, tumor cells underwent stage-specific transcriptomic and metabolic rewiring while growing within an immunosuppressive tumor microenvironment.

### *Plxna2* marks an expanded-like alveolar remodeling state during WT lung organoid formation

**Figure 3:**
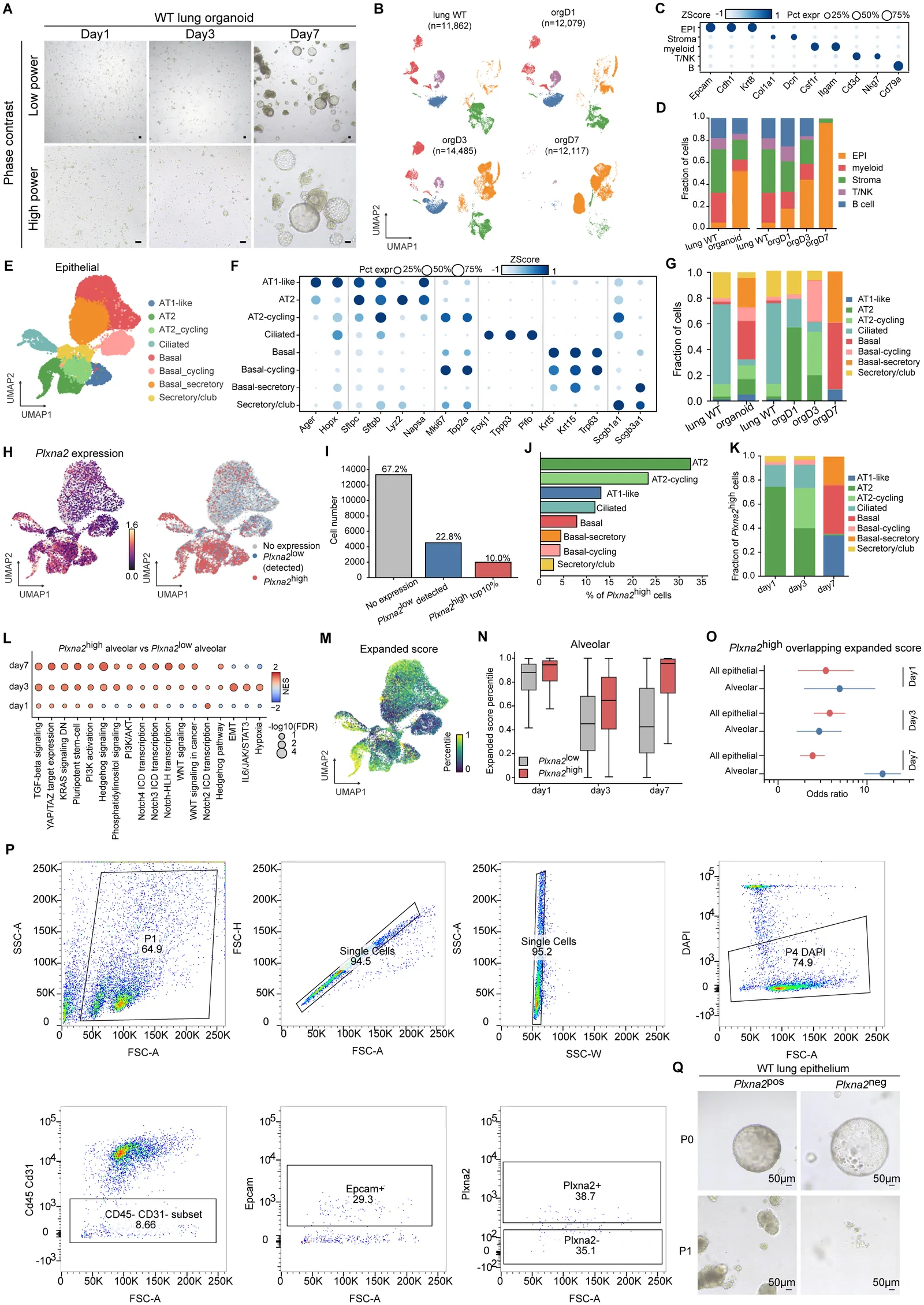
*Plxna2* marks an expanded-like alveolar remodeling state in WT lung organoids. **(A)**, Representative phase-contrast images of WT lung organoids at days 1, 3 and 7 (n = 3 independent donor mice). Scale bar, 50 μm. **(B)**, UMAPs of WT lung and WT lung organoid single-cell datasets across samples. **(C)**, Dot plot of broad lineage markers used for cell-class annotation. **(D)**, Cell type composition of WT lung and WT lung organoid samples. **(E)**, UMAP of reannotated WT epithelial cells. **(F)**, Dot plot of epithelial subtype markers defining AT1-like, AT2, AT2-cycling, ciliated, basal, basal-cycling, basal-secretory and secretory/club states. **(G)**, Epithelial subtype composition across WT lung and organoid time points. **(H)**, UMAPs showing *Plxna2* expression and day-matched *Plxna2*^high^, *Plxna2*^low^ and Plxna2-negative epithelial cells. **(I)**, Number and fraction of WT epithelial cells classified as Plxna2-negative, *Plxna2*^low^ detected or *Plxna2*^high^ top 10%. **(J)**, Epithelial subtype composition of *Plxna2*^high^ cells. **(K)**, Time-point-specific subtype composition of *Plxna2*^high^ epithelial cells. **(L)**, GSEA comparing *Plxna2*^high^ and *Plxna2*^low^ alveolar cells at each organoid time point. **(M)**, Projection of the barcoded expanded-lineage signature onto WT epithelial cells. **(N)**, Expanded-lineage score percentiles in *Plxna2*^high^ and *Plxna2*^low^ alveolar cells across organoid time points. **(O)**, Odds ratios for overlap between *Plxna2*^high^ and expanded-like top 10% cells among all epithelial cells and within the alveolar subset. **(P)**, Representative flow-cytometry gating strategy for isolating *PLXNA2*^pos^ and *PLXNA2*^neg^ epithelial cells from WT mouse lung. **(Q)**, Representative phase-contrast images of organoids derived from sorted *PLXNA2*^pos^ and *PLXNA2*^neg^ WT lung epithelial cells at the initial culture (P0) and after the first passage (P1). *PLXNA2*^pos^ cells retained organoid-forming capacity after passaging, whereas organoid formation from the *PLXNA2*^neg^ population was markedly reduced. Scale bars, 50 μm.

Recent studies suggest that the organoid culture conditions and the alveolar progenitor-like states can influence early *Kras*/*Trp53*-driven LUAD tumorigenesis (49–51). Therefore, we asked whether *Plxna2* could mark a subset of cells in WT lung that might harbor tumor-initiating potential. We derived WT lung organoids using the same culture system as p53K_org. Morphologically apparent WT organoids emerged by day 7 of culture (**Figure 3A**). We then performed 3’ scRNA-seq across WT lung tissue and organoid cultures at days 1, 3 and 7. The pooled dataset contained epithelial, stromal, myeloid, T/NK and B cell compartments (**Figures 3B, C**). At early time points, immune and stromal cells remained abundant, whereas epithelial cells were gradually enriched during culture (**Figure 3D**). Subclustering of epithelial cells identified AT1-like, AT2, cycling AT2, basal, ciliated and secretory/club populations (**Figures 3E, F**). By day 7, organoid cultures contained an increased basal-cell fraction relative to WT lung tissue (**Figure 3G**), indicating culture-associated epithelial selection and remodeling rather than preservation of the starting lung composition.

To determine whether the *Plxna2*-associated state observed in p53K_LC organoids was also present during WT epithelial organoid formation, we examined *Plxna2* expression across days 1, 3 and 7. Plxna2 was detected in approximately 30% of epithelial cells and was preferentially expressed in alveolar-lineage populations (**Figures 3H, I**). *Plxna2*^high^ cells were enriched among AT2, cycling AT2 and AT1-like cells (**Figure 3J**), suggesting that *Plxna2* marks a subset of alveolar-lineage epithelial cells rather than a broadly distributed epithelial state. Although the overall alveolar-cell fraction declined by day 7 as basal-like cells increased, the *Plxna2*^high^ compartment remained relatively biased toward alveolar-lineage cells compared with the total epithelial cells (**Figures 3G, K**).

We next compared *Plxna2*^high^ and *Plxna2*^low^ alveolar cells within each organoid time point. GSEA showed that Plxna2high cells were enriched for programs linked to epithelial plasticity, niche interaction and tissue remodeling, with the strongest and most robust signals at days 3 and 7. These included ECM-receptor interaction, focal adhesion, TGF-β signaling, YAP/TAZ target expression, pluripotent stem-cell transcription, Hedgehog signaling and Notch/WNT-related programs. Day 3 additionally showed enrichment of EMT, hypoxia and IL6/JAK/STAT3 programs, suggesting that Plxna2high alveolar cells transiently acquire a stress-associated remodeling state during organoid growth (**Figure 3L**). These findings fit with recent studies showing that AT2 fate plasticity and alveolar repair states are regulated by CEBPA/DLK1-Notch circuitry and Hippo-YAP/TAZ signaling (52–54).

To test whether the lineage findings can be validated in WT settings, we projected the expansion signature from p53K_LC organoids onto the WT epithelial atlas (**Figure 3M**). Within the alveolar compartment, *Plxna2*^high^ cells showed higher expanded-like scores than *Plxna2*^low^ cells at all three time points, with the strongest difference at day 7 (**Figure 3N**). Similarly, *Plxna2*^high^ cells were enriched among expanded-like top 10% cells in both across all epithelial cells and within the alveolar subset, with a stronger pooled odds ratio in alveolar cells than in the total epithelium (**Figure 3O**). These data indicate that *Plxna2* marks an alveolar remodeling state in WT organoids that shares transcriptional features with the expansion state. We further isolated *Plxna2*^pos^ and *Plxna2*^neg^ cells from WT lung cells by FACS (**Figure 3P**). *Plxna2*^pos^ cells generated organoids that could be maintained across successive passages, whereas the organoid-forming capacity of *Plxna2*^neg^ cells declined and could not be maintained beyond passage 1 (**Figure 3Q**).

### Single-cell spatial transcriptomics links *Plxna2*^high^ epithelial cells to a stromal EMT-like niche

**Figure 4:**
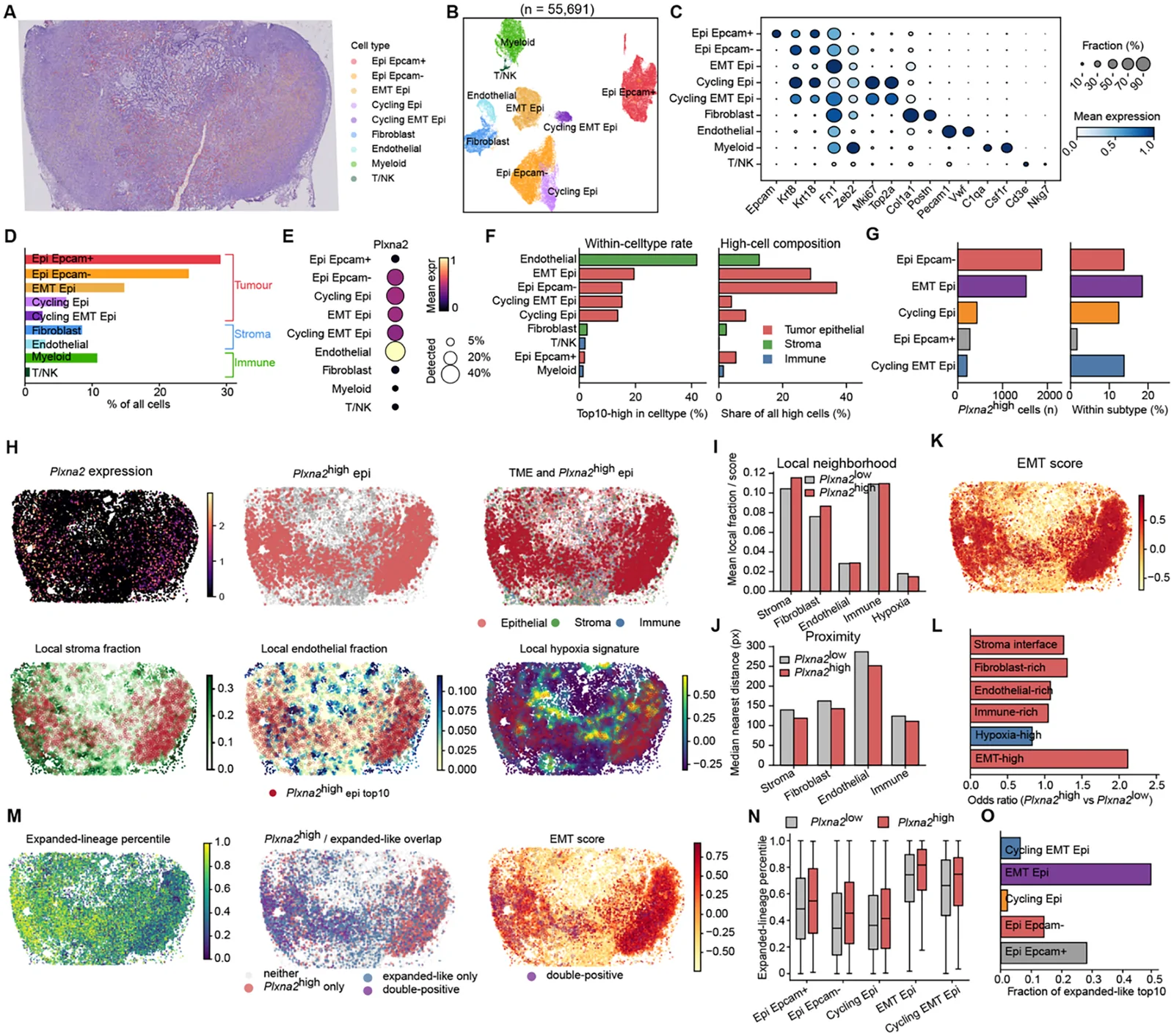
Spatial mapping links *Plxna2*^high^ tumor epithelial cells to an EMT/stromal interface niche. **(A)**, Spatial tumor section with annotated tumor epithelial, stromal and immune cell classes overlaid on histology. **(B)**, UMAP of the spatial single-cell dataset colored by annotated cell type. **(C)**, Dot plot of marker genes used to annotate spatial cell types. **(D)**, Cell-type composition of the spatial tumor section. **(E)**, *Plxna2* expression and detection frequency across spatial cell types. **(F)**, All-cell *Plxna2*^high^ analysis showing the within-cell-type high rate and the contribution of each cell type to the global *Plxna2*^high^ population. **(G)**, Tumor epithelial-only *Plxna2*^high^ analysis showing *Plxna2*^high^ cell counts and within-subtype high rates. **(H)**, Spatial maps of Plxna2 expression, *Plxna2*^high^ epithelial cells, tumor microenvironment cells with *Plxna2*^high^ epithelial cells, local stromal fraction, local endothelial fraction and local hypoxia signature. **(I)**, Local neighborhood fractions or scores comparing *Plxna2*^high^ and *Plxna2*^low^ tumor epithelial cells. **(J)**, Median distance from *Plxna2*^high^ and *Plxna2*^low^ tumor epithelial cells to stromal, fibroblast, endothelial and immune compartments. **(K)**, Spatial projection of the EMT score. **(L)**, Odds ratios for enrichment of *Plxna2*^high^ tumor epithelial cells in stromal, fibroblast-rich, endothelial-rich, immune-rich, hypoxia-high and EMT-high spatial categories. **(M)**, Spatial projection of expanded-lineage percentile, *Plxna2*^high^/expanded-like overlap groups and EMT score. **(N)**, Expanded-lineage percentiles in *Plxna2*^high^ and *Plxna2*^low^ cells within each tumor epithelial subtype. **(O)**, Tumor epithelial subtype composition of expanded-like top 10% spatial cells.

To determine where *Plxna2*^high^ epithelial cells localize *in situ*, we profiled a late-stage tumor section using SeekSpace spatial transcriptomics. SeekSpace enables high-resolution single-cell spatial transcriptomic mapping, allowing transcriptomic cell states to be interpreted within their native tissue architecture (55). In total, we recovered 55,691 spatially resolved cells from the late-stage tumor section, including epithelial, fibroblast, endothelial, myeloid and T/NK compartments (**Figures 4A–C**). Compared with dissociated 5’ scRNA-seq, the spatial dataset contained a larger epithelial fraction, providing a complementary view of epithelial organization within the tumor section (**Figures 4D**, **1G**).

Across all spatially resolved cells, endothelial cells showed the highest *Plxna2* expression and detection frequency, followed by EMT epithelial cells (**Figure 4E**). However, when all *Plxna2*^high^ cells were decomposed by cell type, Epcam- epithelial cells and EMT epithelial cells represented major contributors to the *Plxna2*^high^ population (**Figure 4F**). Likewise, epithelial-only analysis showed that *Plxna2*^high^ tumor cells were concentrated in Epcam- and EMT epithelial states (**Figure 4G**).

We then tested whether the *Plxna2*^high^ epithelial state occupied a distinct tumor microenvironment niche. Projection of *Plxna2* expression and *Plxna2*^high^ epithelial cells onto the tumor section revealed preferential localization near regions with higher local fibroblast fractions (**Figure 4H**). Quantification of local neighborhoods showed that *Plxna2*^high^ epithelial cells had more fibroblast neighbors than *Plxna2*^low^ epithelial cells (**Figures 4I, J**). Moreover, *Plxna2*^high^ epithelial cells were most enriched in EMT-high epithelial regions, followed by fibroblast-rich and tumor-stroma interface regions, whereas hypoxia-high regions were depleted (**Figures 4K, L**). This spatial pattern suggests that *Plxna2*^high^ cells are associated with a stromal EMT-like and fibroblast-enriched niche, a context linked to tumor progression and treatment resistance (56, 57).

Finally, we projected the expansion signature onto the tumor section. *Plxna2*^high^ cells partially overlapped with epithelial cells carrying high expansion scores, suggesting that *Plxna2* marks an enriched but non-exclusive subset of the expanded-lineage state (**Figure 4M**). Within each epithelial subtype, *Plxna2*^high^ cells showed higher expanded-lineage percentile scores than *Plxna2*^low^ cells (**Figure 4N**). The expanded-like top 10% compartment was dominated by EMT epithelial cells, which contributed the largest fraction of expanded-like spatial epithelial cells (**Figure 4O**). These spatial data results place *Plxna2*^high^ epithelial cells within a fibroblast-associated, EMT-enriched tumor niche and support a spatial connection between *Plxna2* expression, epithelial plasticity and the expansion state.

### *PLXNA2* marks a subset of LUAD tumor cells with stemness features

**Figure 5:**
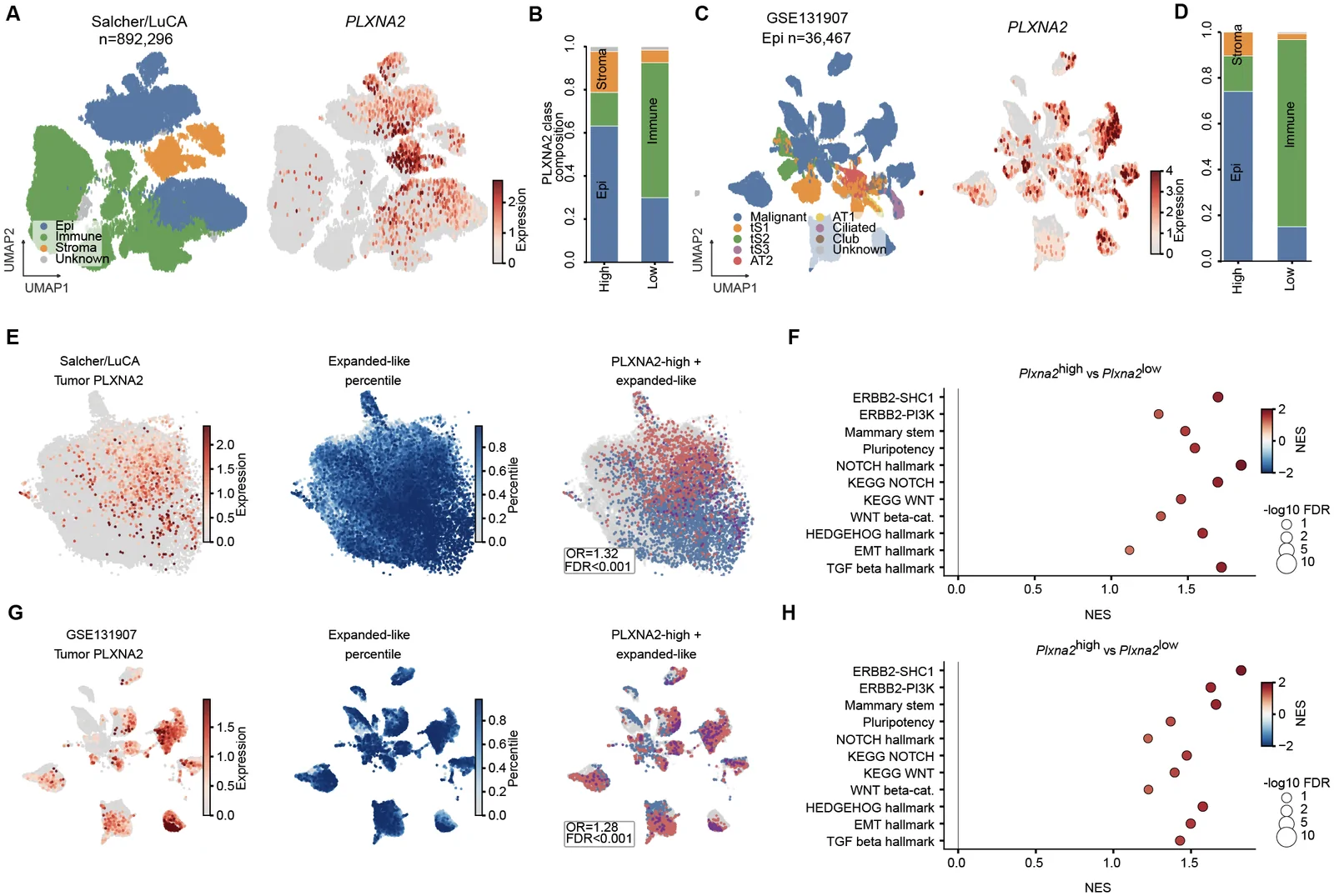
Patient LUAD cohorts validate *PLXNA2*^high^ expanded-like tumor states. **(A)**, UMAP of the Salcher/LuCA lung cancer atlas (58) colored by broad cell class, alongside *PLXNA2* expression across all cells. **(B)**, Broad cell-class composition of *PLXNA2*^high^ and *PLXNA2*^low^ cells in the Salcher/LuCA atlas. **(C)**, UMAP of epithelial cells from GSE131907 (30), alongside *PLXNA2* expression across all cells. **(D)**, Broad cell-class composition of *PLXNA2*^high^ and *PLXNA2*^low^ cells in GSE131907. **(E)**, Salcher/LuCA malignant cells showing *PLXNA2* expression, expanded-lineage percentile and overlap between *PLXNA2*^high^ and expanded-like cells. *PLXNA2*^high^ and expanded-like cells were defined within each malignant sample. **(F)**, Gene-set enrichment analysis of *PLXNA2*^high^ and *PLXNA2*^low^ malignant cells in Salcher/LuCA. **(G)**, GSE131907 malignant epithelial cells showing *PLXNA2* expression, expanded-lineage percentile and overlap between *PLXNA2*^high^ and expanded-like cells. *PLXNA2*^high^ and expanded-like cells were defined within each malignant sample. **(H)**, Gene-set enrichment analysis of *PLXNA2*^high^ and *PLXNA2*^low^ malignant epithelial cells in GSE131907.

We then evaluated whether *PLXNA2* is a potential expansion-state marker in LUAD patient cohorts. We analyzed two LUAD patient cohorts and examined *PLXNA2* expression (30, 58). *PLXNA2* expression was detected mainly in epithelial and stromal compartments, similar to the pattern observed in mouse spatial data (**Figures 5A, C**). After restricting analysis to high-expressing cells, *PLXNA2*^high^ events were mainly malignant epithelial cells (**Figures 5B, D**). To determine whether *PLXNA2*^high^ tumor cells exhibit a preference for the expansion state, we subclustered all malignant cells from the two datasets (**Figures 5E, G**). *PLXNA2*^high^ cells were enriched within the expanded-like compartment across both cohorts (Salcher/LuCA: OR = 1.32, FDR<0.001; GSE131907: OR = 1.28, FDR<0.001; **Figures 5F, H**). These results suggest that the *PLXNA2*^high^ subset shows a biological bias toward the expanded-like state. GSEA of cells showed enrichment of stemness programs in both cohorts. Overall, these analyses support *PLXNA2* as a marker of a subset of LUAD tumor cells with stemness features.

## Discussion

In this study, we established a barcoded *Kras*^G12D^, *Trp53* loss LUAD organoid system that links founder organoid states to subsequent tumor engraftment by combining 15N barcodes and scRNA-seq. This model captured diverse malignant epithelial programs, including two stemness-related states. Paired lineage analysis in baseline organoids nominated *Plxna2* as a candidate marker of expansion states. Expanded lineages then underwent a stage-specific transitions with distinct adaptation programs and metabolic states. WT organoid, spatial transcriptomic and human patient cohort analyses provided convergent support for the *Plxna2*-associated expansion state. These analyses establish a workflow in which combined 15N barcoding and scRNA-seq can nominate markers of early lung cancer initiation.

These findings both confirm and extend the existing literature. The genetic backbone of the model is *Kras*^G12D^ activation and *Trp53* loss, which builds on previous systems showing that lung epithelial progenitors and AT2 organoids can model oncogenic KRAS and diverse LUAD genotypes (7–10). The lineage strategy is also conceptually aligned with expressed-barcode and single-cell lineage-tracing studies that connect transcriptional state to fate, including CellTagging, LARRY, KP-Tracer and spatial lineage-tracing approaches (12–15, 59). The surfaceome-focused prioritization further extends lineage tracing toward experimental actionability, because the candidate markers can be tested by flow cytometry, antibody perturbation or prospective transplantation (38, 60, 61). Still, this triangulation is not equivalent to direct patient lineage tracing or native orthotopic tumor initiation. The patient-cohort validation should therefore be read as convergent support rather than functional proof.

*Plxna2* is a biologically plausible candidate because it encodes a class-A plexin semaphorin receptor, positioning it to influence epithelial state and tumor-niche signaling. Plexin and semaphorin signaling can regulate cytoskeletal organization, adhesion, proliferation, migration, invasion and tumor-stroma interactions (40, 41). In our system, *Plxna2*^high^ cells were enriched among baseline organoid cells belonging to the expanded lineage. These *Plxna2*^high^ cells showed higher MP2 usage, TIC-like and stemness scores. *Plxna2*^high^ cells were also enriched in Notch, WNT, Hippo/YAP-TAZ and TGF-β-related programs. The WT organoid data suggest that this state is also present in normal lung organoids and enriched in AT2 cells. This observation aligns with current literature showing that AT2 plasticity and alveolar progenitor-like states can influence lung repair and tumorigenic competence (51, 52, 62). Spatial data extended this support *in situ*. We found that although *Plxna2* has endothelial background expression, *Plxna2*^high^ epithelial cells were enriched within Epcam-low or EMT-like epithelial compartments and localized near fibroblast-rich tumor-stroma interfaces. Human LUAD cohort analyses further support *PLXNA2* as a candidate marker for studying early tumor expansion.

Several limitations should be considered. First, the current evidence supports *PLXNA2* as a candidate marker of an expansion-associated epithelial state but does not establish it as a definitive cancer stem-cell marker. Although *Plxna2*^pos^ WT lung cells showed sustained organoid-forming capacity, prospective transplantation of *Plxna2*^pos^ and *Plxna2*^neg^ cells from *Trp53 loss*, *Kras*^LSL-G12D^ organoids has not been performed. In addition, the organoids were established from whole-lung tissue, and Cre-mediated genetic activation was not restricted to a specific epithelial subtype. Future studies using purified AT2 organoids and orthotopic or autochthonous models will be required to establish the cell of origin and directly test tumor-initiating capacity (10, 63–65). Also, subcutaneous transplantation does not reproduce the native alveolar architecture and may alter stromal recruitment, immune infiltration, and results in different epithelial state. Further studies using orthotopic transplantation or autochthonous model will be required to validate our findings (66).

Second, lineage recovery was limited with barcodes recovered from 4.53% of epithelial cells. Incomplete recovery may have resulted from limited or heterogeneous miniP activity, altered transcription activity, epigenetic silencing of the lentiviral cassette, and stochastic loss of less abundant transcripts (67–71). To reduce this effect, we restricted the primary analysis to high-confidence barcode assignments. Nevertheless, the workflow itself provides a useful paradigm. Combining barcoded organoid founder populations with scRNA-seq can nominate candidate markers. Candidate markers can then be filtered or validated through WT organoid data, spatial tumor context and patient multi-omics cohorts. Because this strategy emphasizes cell-surface proteins, it creates a practical bridge from discovery to FACS-based validation, mechanistic perturbation and, eventually, antibody-based therapeutic targeting.

In order to improve the recovery rate, barcode and EGFP transcript abundance could be quantified by RT-qPCR and amplicon sequencing at defined stages, including after FACS enrichment, before transplantation, and in early- and late-stage tumors, to determine whether reporter expression declines during culture or tumor progression. Targeted PCR could also be improved by optimizing conditions to increase the sequencing depth. For future improvement of the system, promoter activity could be compared across promoters in lung organoids, and the lineage barcode could be embedded within the 3′ untranslated region of a highly expressed polyadenylated reporter transcript. These experiments would provide a direct basis for distinguishing incomplete integration, transcriptional silencing, and technical failure of RNA-level barcode detection.

Overall, this work suggests that LUAD growth can arise from a pre-existing epithelial state that is selected and reshaped by the *in vivo* tumor microenvironment. *Plxna2*^high^ cells emerged as an experimentally tractable candidate marker in this context. They are enriched during engraftment, persist as an expansion state in WT alveolar organoids, and localize in tumors to EMT and stromal-interface niches. This study provides a discovery framework for finding surface markers of epithelial states with tumor-expansion potential. Future functional validation will determine whether PLXNA2 is an actionable regulator of the transition from transformed organoid cell to expanding tumor lineage.

## Methods

### Mouse lines

The Institutional Animal Care and Use Committee of Renji Hospital evaluated and approved all animal protocols prior to the commencement of the study (RJ2022-0104). Experiments primarily utilized C57BL/6JGpt mice (hereafter C57BL/6; GemPharmatech Co., Ltd, Strain NO. N000013, MGI: 6314664). Cyagen Biosciences was responsible for the generation and breeding of the *Trp53*^fl/fl^, *Kras*^LSL-G12D^ transgenic lines on the C57BL/6 background.

### Lung organoid derivation and culture

Pulmonary tissues required for organoid generation were collected from WT C57BL/6 or *Trp53*^fl/f^, *Kras*^LSL-G12D^ mice immediately after euthanasia by cervical dislocation. Initially, the lungs were rinsed in phosphate-buffered saline (PBS, BasalMedia, Cat# B320KJ) and minced with scissors. The primary digestion used 10 ml Dulbecco’s modified Eagle’s medium (DMEM, BasalMedia, Cat# L110KJ) supplemented with a 1:10 dilution of collagenase/hyaluronidase (STEMCELL Technologies, Cat# 07912) and 1 mg ml^-1^ dispase (STEMCELL Technologies, Cat# 07913) incubating at 37 °C for 30 min. Cells were then pelleted at 300 × g for 5 min, the supernatant was removed, and a secondary dissociation was performed with 5 ml of TrypLE (Gibco, Cat# 12604021) for 10 min at 37 °C. The mixture was passed through a 40-µm mesh and centrifuged. Then, aliquots of 100–10,000 cells were resuspended in Ceturegel Matrix (YEASEN, Cat# 40192ES10), seeded into 24-well plates as 40 µl domes, and overlaid with 400 µl of medium per well. Two commercially available media were used for non-transformed and transformed organoids, respectively. Normal lung organoids derived from WT C57 mice and untransformed organoids derived from *Trp53*^fl/f^, *Kras*^LSL-G12D^ mice were cultured in mouse lung epithelial organoid culture medium (OneTAR, Cat. No. OneTar-mLC-N100). Following Cre recombinase transduction, which activated the *Kras*^G12D^ allele and deleted Trp53, the transformed organoids were cultured in lung adenocarcinoma organoid culture medium (Vazyme, Cat. No. GM204). Organoids were maintained at 37 °C in a humidified incubator with 5% CO_2_, and fresh medium was supplied every third day for up to four weeks.

Organoids were passaged at a 1:3 ratio. Briefly, after medium aspiration, the Ceturegel Matrix was dissolved by adding 200 µl of organoid recovery solution (YEASEN, Cat# 41421ES60). The mixture was kept on ice for 15–30 min and gently pipetted to fragment the organoids. After 5 min centrifugation at 300 × g, the resulting cell pellets were seeded into new matrix. Cryopreservation was performed using organoid freezing medium (YEASEN, Cat# 41422ES60).

### Lentiviral production

We used an in-house lentiviral vector v8-Puro-IRES-Cre. Within the lentiviral transfer cassette, the human EF1α promoter drives expression of a puromycin-resistance–IRES–Cre cassette, followed by the WPRE. The transfer cassette is located between the 5′ and 3′ long terminal repeats (LTRs) (**Supplementary Figure 1A**).

To generate lentiviral particles, human embryonic kidney 293T cells were maintained at 37 °C with 5% CO_2_ in DMEM containing 10% fetal bovine serum (FBS; ExCell, Cat# FSP500) and 100 U ml^-1^ penicillin/streptomycin (BasalMedia, Cat# S110JV). Cells were co-transfected with the 15N lentiviral library or Puro-IRES-Cre and appropriate helper plasmids. Transfection was initiated when cells reached 70%–80% confluence. 48 hours after transfection, the culture media was harvested. Initial low-speed centrifugation was used to clear cellular debris, followed by ultracentrifugation to pellet and concentrate the viral particles. These pellets were subsequently resuspended in fresh basal medium, divided into aliquots, and cryopreserved at −80 °C.

### Organoid infection

After 3 weeks of culture, p53K_org organoids were transduced with concentrated v8-Puro-IRES-Cre lentivirus. Organoids were exposed to the lentiviral suspension and subjected to spinoculation at 100 × g for 4 h. The viral supernatant was then removed, and the organoids were washed with sterile PBS, resuspended in Ceturegel Matrix, and returned to organoid culture. At 3 days after transduction, puromycin (YEASEN, Cat. No. 727136ES01) was added at 2ug ml^-1^ for 3 consecutive days. Puromycin-resistant organoid cells were subsequently recovered and maintained in lung adenocarcinoma organoid culture medium.

Two weeks after Puro-IRES-Cre transduction, p53K_LC_org organoids were transduced with the concentrated 15N barcode lentiviral library at a multiplicity of infection (MOI) of 1. To maximize viral entry, the mixture underwent spinoculation at 100 × g for 4 h. Following this, the viral supernatant was aspirated, and organoids were washed with sterile PBS to eliminate unbound virions. Finally, the infected organoids were resuspended in a Ceturegel Matrix, seeded, and cultured under standard conditions.

### Genomic validation of Cre-mediated recombination

Genomic DNA was extracted from organoids using a DNA extraction kit (Vazyme, Cat. No. DC102-01). PCR amplification was performed using 2× Phanta UniFi Master Mix (Dye Plus) (Vazyme, Cat. No. P526), according to the manufacturer’s instructions. Primer sequences were as follows: Trp53-exon3-F, 5′-CCATCACCTCACTGCATGGA-3′, Trp53-exon3-R, 5′-TGTCCCAGACTGCAGGAAGC-3′, Kras-g12-F, 5′-TAATCTTGTGTGAGACATGT-3′, and Kras-g12-R, 5′-CTGCCGTCCTTTACAAGCGC-3′. The PCR product was submitted to Tsingke Biotechnology Co., Ltd. (Beijing, China) for Sanger sequencing.

### Subcutaneous tumor inoculation

At 3 days after passage, the organoids were recovered from the Ceturegel Matrix as described above and mechanically dissociated into small organoid fragments. The fragments were collected by centrifugation and resuspended in a 1:1 (v/v) mixture of PBS and Ceturegel Matrix. Organoid fragments recovered from approximately eight matrix domes were injected subcutaneously into the dorsal flank of each 6–8 week male C57BL/6 mouse in a total volume of 100 μl. Tumor dimensions were measured using calipers. Tumor volume was calculated as 0.5 × length × width^2^. Mice were euthanized when the tumor volume reached 1500 mm^3^. Tumors were collected at 4 weeks after inoculation for early-phase and at 6 weeks after inoculation for late-phase.

### Flow cytometry

#### EGFP-based fluorescence-activated cell sorting

Following transduction with the 15N barcode–EGFP lentiviral library, p53K_LC_org organoids were dissociated into single cells as described above. The cell suspension was neutralized with HBSS supplemented with 2% FBS, passed through a 70-μm cell strainer, and centrifuged. The resulting cell pellet was resuspended in PBS containing 1% BSA and maintained on ice until sorting.

Cells were sorted using a BD FACSAria II cell sorter (BD Biosciences). Intact cells were first selected according to FSC-A and SSC-A, followed by FSC-A and FSC-H gating to exclude doublets and retain singlets. The EGFP-positive gate was defined using untransduced p53K_LC_org cells as a negative control. EGFP-positive cells were collected, embedded in Ceturegel Matrix, and cultured in lung adenocarcinoma organoid culture medium for 3 weeks before subcutaneous transplantation.

#### Plxna2-based sorting of WT lung epithelial cells

WT mouse lung cells were dissociated into single-cell suspensions as described above. The suspensions were neutralized with HBSS supplemented with 2% FBS, passed through a 70-μm cell strainer, and centrifuged. The resulting cell pellets were resuspended in PBS containing 1% BSA.

Cells were subsequently incubated with an anti-PLXNA2 antibody (Cell Signaling Technology, Cat. No. 5658; clone D42B5) for 30 minutes on ice. After washing, PLXNA2 labeling was detected using a FITC-conjugated anti-rabbit IgG secondary antibody (BioLegend, Cat. No. 406403). Cells were then stained with PE anti-mouse CD45 (BioLegend, Cat. No. 103106), PE anti-mouse CD31 (BioLegend, Cat. No. 102408), and PerCP/Cyanine5.5 anti-mouse CD326 (EpCAM) (BioLegend, Cat. No. 118220) for 30 min on ice in the dark. DAPI was added before sorting to exclude nonviable cells. Sorted cells were embedded in Ceturegel Matrix and cultured under identical WT lung organoid conditions.

#### Flow cytometric analysis of immune cells

Cells were dissociated as described above. The cell suspension was neutralized using HBSS supplemented with 2% FBS. Following filtration through a 70-µm strainer and subsequent centrifugation, the resulting cell pellets were resuspended in PBS enriched with 1% bovine serum albumin (BSA). To prevent nonspecific Fc receptor interactions, the cells underwent a 15-minute incubation on ice with an anti-mouse CD16/32 antibody. This was followed by a 30-minute staining period on ice in the dark using a specific fluorochrome-conjugated antibody cocktail. The antibody panel included: APC anti-mouse CD45 (BioLegend, cat# 103111), PE/Cyanine7 anti-mouse CD45 (BioLegend, cat# 147703), Alexa Fluor^®^ 700 anti-mouse CD3 (BioLegend, cat# 100215), FITC™ anti-mouse CD4 (BioLegend, cat# 100405), Pacific Blue anti-mouse CD8a (BioLegend, cat# 100728), PE anti-mouse CD279™ (PD-1) (BioLegend, cat# 135205), Pacific Blue anti-mouse/human CD11b (BioLegend, cat# 101223), and APC anti-mouse CD274 (B7-H1, PD-L1) (BioLegend, cat# 124311). Post-staining, the cells were washed twice with PBS and exposed to DAPI (1:2000 dilution) in FACS buffer for 10 minutes to facilitate live/dead cell differentiation. Finally, flow cytometric data were acquired on a BD LSR Fortessa instrument (BD Biosciences) and analyzed using FlowJo software.

### Histology and immunohistochemistry

To prepare specimens for histopathological and immunohistochemical analyses, human or murine tissues were harvested, washed with PBS, and fixed overnight in 4% paraformaldehyde at 4 °C. Following dehydration and paraffin embedding, the FFPE blocks were sectioned at a thickness of 5 µm. Routine hematoxylin and eosin (H&E) staining was performed after the sections were deparaffinized in xylene and rehydrated through a graded ethanol series.

For immunohistochemical profiling, heat-induced antigen unmasking was performed in a pressure cooker with Tris-EDTA buffer (pH 9.0). To prevent nonspecific binding and eliminate endogenous peroxidase activity, 5% bovine serum albumin and 3% H2O2 were applied, respectively. Slides were then incubated at 4 °C overnight with the following primary antibodies: anti-CK8 (1:200, Abcam, Cat# ab53280), anti-CK5 (1:200, Abcam, Cat# ab52635), and anti-Ki67 (1:200, Abcam, Cat# ab16667). Signals were visualized using an HRP-conjugated secondary antibody (1:200, Abcam, Cat# ab6721) coupled with DAB chromogen (YEASEN, Cat# 36302ES01), followed by nuclear counterstaining with hematoxylin.

### 5’ and 3’ single-cell RNA sequencing

#### Cell preparation

Harvested tissues were washed in ice-cold RPMI1640 and processed via the Multi Tissue Dissociation Kit 2 (Miltenyi Biotec, Cat# 130-110-203), supplemented with additional DNase treatment for highly viscous samples. Red blood cells were then depleted (Miltenyi Biotec, Cat# 130-094-183). Cell counts and viability were evaluated using AO/PI staining on a Countstar Rigel S2 Cell Analyzer. Whenever initial viabilities were suboptimal, debris and dead cells were selectively removed (Miltenyi Biotec, Cat# 130-109–398 and 130-090-101). The resulting single-cell suspensions were washed twice with RPMI1640 and resuspended at 1 × 10^6^ cells ml^-1^ in a 0.04% BSA-PBS solution.

#### 5’ scRNA-seq

The SeekOne^®^ DD Single Cell 5’ library preparation kit (SeekGene, Cat# K00501) was used for scRNA-seq library generation. Cells and RT reagents were co-encapsulated with Gel Beads and Partitioning Oil within a SeekOne^®^ DD Chip S3. Reverse transcription was performed inside the droplets at 42 °C (90 min) and heat-inactivated at 85 °C (5 min). After droplet disruption and purification, the cDNA was PCR-amplified, fragmented, end-repaired, A-tailed, and ligated to sequencing adapters. Cell barcodes and UMIs were integrated during a final indexing PCR step. Libraries were purified via VAHTS DNA Clean Beads (Vazyme, N411-01) and assessed using Qubit (Thermo Fisher Scientific, Q33226) and a Bio-Fragment Analyzer (Bioptic, Qsep400). Sequencing was performed on the GeneMind SURFSeq 5000 (PE150 mode).

#### 3’ scRNA-seq

Single-cell RNA-seq libraries were generated using the SeekOne^®^ MM Single Cell 3’ library preparation kit. Cell suspensions were first introduced into the flow channel of the SeekOne^®^ MM chip, where gravity drove deposition into 170,000 discrete microwells. After uncaptured cells were aspirated, Cell Barcoded Magnetic Beads (CBBs) were loaded and magnetically guided into the microwells. After removal of surplus CBBs, *in situ* cell lysis was performed, allowing liberated RNA to be captured by the co-localized CBB. To tag cDNA with bead-specific cell barcodes, reverse transcription was carried out at 37 °C for 30 min. Unextended bead-bound primers were digested with Exonuclease I. Next, the barcoded cDNA was hybridized with random primers containing the Read 2 SeqPrimer sequence at their 5’ ends, driving synthesis of a second DNA strand with the 3’ cell barcode. These second-strand products were thermally denatured from the CBBs, purified and PCR-amplified. Post-amplification cleanup was performed before full-length sequencing adapters and sample indices were added by indexed PCR. Final libraries were purified using SPRI beads, quantified by qPCR (KAPA Biosystems, KK4824), and sequenced on either an Illumina NovaSeq 6000 (PE150 mode) or a DNBSEQ-T7 platform (PE100 mode).

### Preprocessing and quality control of scRNA-seq data

Initial quality control of the raw sequencing reads was performed with Fastp (72) to eliminate low-quality bases and primer sequences. Gene expression matrices were generated by processing and aligning the data to the mouse GRCm38 reference genome via SeekOne^®^ Tools.

Downstream scRNA-seq analysis was performed with Scanpy v1.11.5 (73) in Python 3.11.14. Cell Ranger output matrices were imported via scanpy.read_10x_mtx and merged. We derived QC metrics using scanpy.pp.calculate_qc_metrics, identifying mitochondrial and ribosomal features via mt- and Rps/Rpl prefixes, respectively. Doublets were scored per sample with Scrublet v0.2.3 (74). Baseline filtering retained cells expressing 200–7,000 genes and ≤50,000 UMIs, with ≤15% mitochondrial content and Scrublet scores ≤0.25. Samples were outer-joined, and raw counts were backed up. Following count normalization and log1p transformation, HVGs were identified using the Seurat v3 method on raw counts, excluding mitochondrial/ribosomal genes (75). Data were scaled and PCA was performed. Harmony was used on PCs for batch correction across samples (76). Finally, we constructed a nearest-neighbor graph, performed UMAP embedding, and applied global Leiden clustering.

### InferCNV analysis

InferCNVpy (v0.6.1) was applied to raw UMI counts using the matching cell, gene, annotation, and mm10 genomic-position files (77). CNV expression deviations were inferred using inferCNVpy (infercnvpy.tl.infercnv), with host TME cells designated as the reference population. Key parameters were set as follows: window_size=100, step=10, lfc_clip=3, dynamic_threshold=1.5, and chunksize=5000. inferCNVpy calculates expression relative to the reference after log-fold-change transformation, smooths values across sliding genomic windows, and applies dynamic-threshold denoising to remove noise. For each cell, CNV burden was quantified as the mean squared deviation across all inferred genomic windows. Chromosome profiles represent the mean window-level deviation across cells within each group.

### Targeted 15N barcode sequencing

To selectively enrich 15N lineage barcodes from the initial single-cell cDNA library, we used a nested PCR enrichment strategy. Briefly, the first round of enrichment PCR was performed with 5 μL of cDNA product and specific primers for 12 cycles. The resulting amplicons were purified using a two-step DNA magnetic bead selection process (0.5× followed by 1.0× ratio) to isolate the target fragments. Next, 23 μL of purified product was used for a second enrichment PCR for 10 cycles with the same primer pair, followed by an identical two-step bead purification. For final sequencing library construction, 10 μL of the enriched cDNA was amplified for 6–10 cycles using N5 and N7 indexing primers. The final indexed library was purified with a 0.9× bead ratio, yielding targeted fragments of approximately 350–400 bp ready for sequencing.

### Lineage calling and categorization

The lentiviral barcode cassette contained a random 15-nucleotide lineage barcode flanked by the upstream constant sequence GAAAGCTAAG and the downstream ACCGGT anchor. Barcode library reads were processed into tables containing the recovered barcode sequence (hereafter referred to as the spacer), single-cell barcode (barcode), unique molecular identifier (UMI), and number of reads. Cell barcodes recovered from the spacer library were matched to cells in the corresponding gene expression matrix. Putative sequencing errors were corrected using the directional adjacency method implemented in UMI-tools (78). The corrected barcode sequences were then assigned lineage identifiers (L0001, L0002 and so forth). For each cell, the corrected lineage barcode with the greatest UMI support was designated the dominant lineage assignment. Among the 1,092 epithelial cells with a recovered lineage barcode, 40 cells (3.66%) contained more than one corrected barcode. In all 40 cells, the secondary barcode was supported by only one or two UMIs; therefore, these cells were assigned to the lineage represented by the dominant barcode.

Lineage fate was assigned using a two-axis framework adapted from prior studies (15, 79, 80). First, we assessed engraftment by absolute lineage presence across organoid, early-stage tumor and late-stage tumor epithelial compartments. Second, we assessed competitive recovery by comparing each lineage’s tumor fraction with its baseline organoid fraction using pseudocount-stabilized log2 fold change, Fisher exact tests and binomial sampling probabilities. Lineages with sustained or enriched tumor recovery were classified as expanded. Lineages detected in tumor but significantly depleted relative to baseline were classified as diminished. Baseline-abundant lineages with absent or near-absent tumor recovery and low dropout probability were classified as failed. Ambiguous or low-depth lineages were classified as underpowered and excluded from primary founder-state contrasts.

### Spatial transcriptomics

Spatial transcriptomics was performed using SeekSpace as previously described (55, 81). Stamp-seq spatial libraries were constructed using the SeekSpace^®^ Single-Cell Spatial Transcriptome-seq Kit (K02501-08). Briefly, 10-μm fresh-frozen cryosections were mounted onto SeekSpace^®^ chips pre-arrayed with 32-nt random ssDNA spatial barcodes. Following mild tissue melting (37 °C, 90 s) and *in situ* spatial barcode cleavage via USER^®^ enzymes, the sections were fixed, fluorescently imaged, and mechanically homogenized to yield single-nucleus suspensions (100,000–200,000 nuclei/sample).

For multiplexed library preparation, isolated nuclei were aliquoted for initial reverse transcription with sample-specific multiplex barcodes. The pooled nuclei were then combined with bridge-oligos and T4 ligase, and encapsulated in emulsion droplets with Barcoded Hydrogel Beads (BHBs) via the SeekOne^®^ Digital Droplet System. Following intra-droplet ligation (20 °C, 60 min) and proteinase K-mediated decrosslinking, the released templates underwent secondary reverse transcription and pre-amplification. The products were subsequently split to construct parallel cDNA and spatial barcode libraries, which were finalized by indexed PCR, purified, and quantified prior to sequencing.

### Analysis of spatial transcriptomics

Spatial transcriptomics data from the tumor section were analyzed in Python 3.11.14 using Scanpy v1.11.5. The SeekSpace filtered feature-barcode matrix was imported with scanpy.read_10x_mtx using gene symbols, and gene names were made unique. Cell coordinates from cell_locations.tsv.gz were aligned to the AnnData barcode order and stored in adata.obsm[“spatial”].

Quality-control metrics were computed for each cell. Cells were retained if they contained at least 200 and at most 6,000 detected genes and less than 25% mitochondrial counts. Genes detected in fewer than 10 cells were removed. Cell-type annotations were assigned by combining Leiden clusters, cluster marker genes and canonical lineage markers. Differential marker genes for Leiden 0.5 clusters were identified by Wilcoxon rank-sum testing on the log-normalized matrix.

Aligned H&E and DAPI images were used for spatial visualization. Cell coordinates were plotted in the original chip coordinate system, with a chip extent of 56,500 by 20,434 pixels. All spatial distance measurements are therefore reported in native chip-pixel units. For visualization, expression and signature maps were plotted at the cell coordinate level and, where appropriate, color scales were clipped to robust percentiles to reduce the influence of extreme values.

Spatial neighborhoods were quantified using a k-nearest-neighbor graph built from x-y cell coordinates with scipy.spatial.cKDTree. For each cell, the 50 nearest neighboring cells, excluding the index cell itself, were used to compute local fractions of non-tumor, stromal, fibroblast, endothelial, immune, myeloid and T/NK cells. Nearest-neighbor distances to each broad compartment or cell type were also calculated in chip-pixel units. Hypoxia and EMT-related local scores were calculated as the mean score among the same 50 neighboring cells. For categorical niche enrichment, high-neighborhood categories were defined by the top quartile of each local metric within tumor epithelial cells.

### Consensus non-negative matrix factorization

cNMF was used to identify recurrent epithelial gene-expression programs. The analysis was performed using cNMF v1.7.1 (20, 82). cNMF was applied to all cleaned epithelial cells. Before cNMF, epithelial cells flagged as high-confidence non-epithelial contaminants or doublets were removed. The final cNMF input contained 24,104 epithelial cells, including 21,450 organoid cells, 1,402 early tumor cells and 1,252 late tumor cells.

Raw counts were used as cNMF input. To prevent technical or housekeeping axes from dominating the factorization, mitochondrial genes, ribosomal genes, and hemoglobin genes were removed before factorization. cNMF was run with 2,000 high-variance genes and 100 independent NMF iterations per rank. Candidate ranks from K = 4 to K = 12 were evaluated using the cNMF rank-selection diagnostics. K = 5 was selected because it provided the highest silhouette score among the evaluated ranks while retaining a compact and biologically interpretable program structure. Consensus programs were generated with a local density threshold of 0.5.

### Gene set enrichment analysis

*Plxna2*^high^ cells were defined as the top 10% of *Plxna2*-expressing baseline organoid epithelial cells, with all remaining baseline organoid epithelial cells used as the low/reference group. Gene-set scores were calculated per cell using scanpy.tl.score_genes in Scanpy (73), and high-low differences were tested using Mann-Whitney U tests with Benjamini-Hochberg correction. GSEA was performed using gseapy (83, 84) against the MsigDB database (85).

### Surfaceome marker selection

Candidate surface markers were nominated from baseline organoid cells whose barcodes mapped to either expanded or failed lineages. Genes were first ranked by expanded-versus-failed expression differences and then intersected with a strict surfaceome annotation based primarily on mouse UniProt (86) subcellular localization and topology, with the Human Protein Atlas (HPA) (60) and Cell Surface Protein Atlas (CSPA) (61) used as supporting resources. For each surface candidate, we quantified cell-level expression enrichment, marker-high enrichment for expanded fate, lineage-level effect size, correlation with expansion-associated MP programs, and specificity relative to the L0001 lineage. Candidate markers were FACS-ready surface proteins with marker-high enrichment for expanded fate. Final core candidates were selected from the list after manual triage for extracellular accessibility, antibody feasibility, marker broadness, and biological interpretability.

### Public dataset validation

Organoid-derived MPs were validated in public human lung scRNA-seq cohorts (30, 31) and public autochthonous KP model datasets (GSE277777 (34) and GSE154989 (33)) using two complementary strategies: direct gene-set projection and supervised phenotype transfer.

For direct projection, the top 50 genes from each of the five organoid-derived cNMF programs were used as MP signatures where available. Per-cell MP activity was calculated with scanpy.tl.score_genes using signature genes. Mean expression and rank-based signature scores were also calculated as robustness summaries.

To complement module-score projection, we performed a Scissor-based supervised phenotype-transfer analysis (32). For each MP, cells with usage above the 80th percentile were used to construct the positive phenotype and cells below the 50th percentile were used to construct the negative phenotype. Expression profiles were aggregated into pseudo-bulk profiles by sample. External single cells were restricted to epithelial cells and sampled to a maximum of 2,500 cells per dataset and MP. Pseudo-bulk and external single-cell matrices were restricted to shared MP signature genes and quantile-normalized jointly. Scissor 2.0 was run with a binomial phenotype model, alpha values of 0.7, 0.8 and 0.9, and cutoff 0.15. Cells were classified as Scissor-positive, Scissor-negative or neutral.

### Statistics and reproducibility

Statistical analyses were performed using Python (v3.11.14), R (v4.3.0) and GraphPad Prism (v10.0). Statistical tests used in this study are specified in the corresponding figure legends. *P* < 0.05 was considered statistically significant. The number of biological replicates for each experiment is reported in the figure legends. Single-cell RNA-seq analysis was processed using established workflows and quality control criteria as described in the Methods.

## Data Availability

The raw sequence data reported in this paper have been deposited in the Genome Sequence Archive (87) in National Genomics Data Center (88), China National Center for Bioinformation/Beijing Institute of Genomics, Chinese Academy of Sciences (GSA: CRA043819) that are publicly accessible at https://ngdc.cncb.ac.cn/gsa.
