# Supplementary material for: Single-cell lineage tracing in tumor organoids identifies expansion states depicting the origin and progression of lung adenocarcinoma
Source: Front Immunol. 2026 Sep 7;17:1903250. doi: 10.3389/fimmu.2026.1903250 (PMC13590773; doi:10.3389/fimmu.2026.1903250)
Supplement: Supplementary file 1 [file DataSheet1.pdf]

Supplementary Materials

Single-cell lineage tracing in tumor organoids identifies expansion states depicting the origin and progression of lung adenocarcinoma

Wutao Chen[1,5], Peng Li[1,5], Hejian Zhang[4,5], Yumeng Sheng[1,5], Guoliang Yang[4,*], Jing Hu[3,*], Bin Yu[1,2,6,*]

[1]State Key Laboratory of Systems Medicine for Cancer, Shanghai Cancer Institute, Renji Hospital, School of Medicine, Shanghai Jiao Tong University, Shanghai 200127, China

[2]Shanghai Key Laboratory for Cancer Systems Regulation and Clinical Translation (CSRCT), Shanghai 200127, China

[3]Department of Anesthesiology, Renji Hospital, Shanghai Jiao Tong University School of Medicine, Shanghai 200127, China

[4]Department of Urology, Renji Hospital, Shanghai Jiao Tong University School of Medicine, Shanghai, China

[5]These authors contributed equally

[6]Lead Contact

[*]Correspondence: hujing4648@126.com (J.H.); ygl0511@126.com (G.L.Y.); yubinrenji@outlook.com (B.Y.)

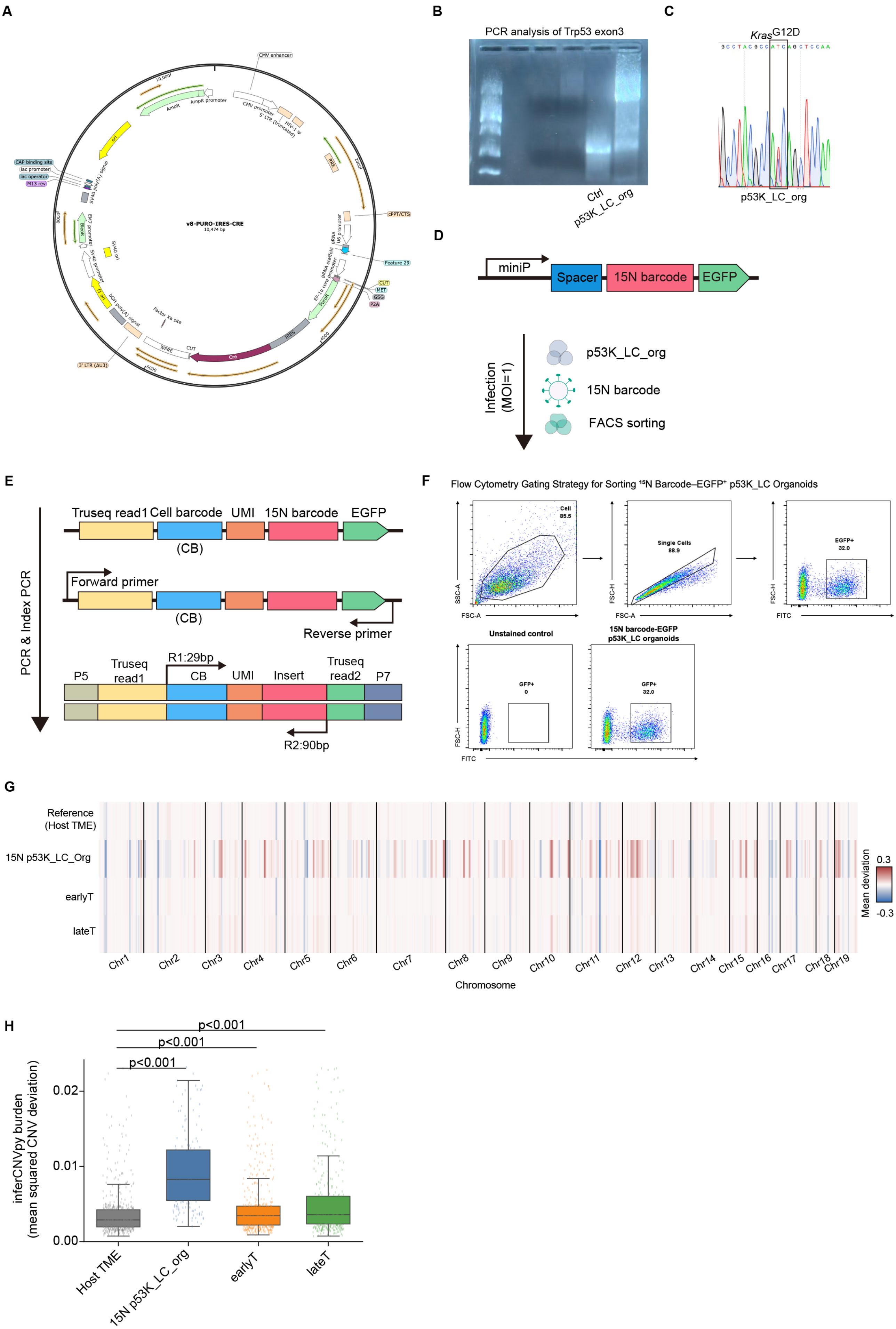
A
v8-PURO-IRES-CRE
10,474 bp
CMV enhancer
CMV promoter
AmpR promoter
AmpR
ori
RRE
cPPT/CTS
U6 promoter
Feature 29
CUT
MET
GSG
P2A
PuroR
IRES
Cre
WPRE
Factor Xa site
bGH poly(A) signal
SV40 promoter
EM7 promoter
BleoR
SV40 ori
SV40 poly(A) signal
CAP binding site
lac promoter
lac operator
M13 rev
3' LTR (ΔU3)
B
PCR analysis of Trp53 exon3
Ctrl
p53K_LC_org
C
*Kras*G12D
p53K_LC_org
D
miniP
Spacer
15N barcode
EGFP
Infection (MOI=1)
p53K_LC_org
15N barcode
FACS sorting
E
PCR & Index PCR
Truseq read1
Cell barcode (CB)
UMI
15N barcode
EGFP
Forward primer
Reverse primer
P5
Truseq read1
R1:29bp
CB
UMI
Insert
Truseq read2
P7
R2:90bp
F
Flow Cytometry Gating Strategy for Sorting 15N Barcode–EGFP+ p53K_LC Organoids
Cell 85.5
Single Cells 88.9
EGFP+ 32.0
SSC-A
FSC-A
FSC-H
FITC
Unstained control
GFP+ 0
15N barcode-EGFP p53K_LC organoids
GFP+ 32.0
G
Reference (Host TME)
15N p53K_LC_Org
earlyT
lateT
Chr1
Chr2
Chr3
Chr4
Chr5
Chr6
Chr7
Chr8
Chr9
Chr10
Chr11
Chr12
Chr13
Chr14
Chr15
Chr16
Chr17
Chr18
Chr19
Chromosome
Mean deviation
0.3
-0.3
H
p<0.001
p<0.001
p<0.001
inferCNVpy burden (mean squared CNV deviation)
0.00
0.01
0.02
Host TME
15N p53K_LC_org
earlyT
lateT

Supplementary Figure 1. Lineage barcoding strategy and meta-program annotation

A, Schematic of the Puro-IRES-Cre vector.

B, PCR analysis of Trp53 exon 3 before and after Cre-mediated recombination. Genomic DNA was isolated from control organoids before Cre transduction (Ctrl) and *Kras*^{G12D}, Trp53-deficient organoids after Cre transduction (p53K_LC_org).

C, Sanger sequencing analysis of the Kras codon 12 region in p53K_LC_org using a reverse primer. The highlighted ATC represents the GAT codon on the coding strand.

D, Schematic of the 15N barcode-EGFP reporter and infection workflow used to label p53K_LC organoids.

E, PCR, indexing and sequencing strategy for recovery of cell barcodes, UMIs and 15N lineage barcodes from single-cell libraries.

F, Flow-cytometry gating strategy for enrichment of 15N barcode–EGFP-positive p53K_LC_org cells.

G, Heatmap of mean inferCNVpy deviation across genomic windows for the host TME reference, organoid, early tumor (earlyT), and late tumor (lateT) epithelial cells.

H, Distribution of cell-level CNV burden. Each dot denotes one cell. *P* value was calculated using Mann-Whitney U test with BH-FDR correction, two-sided.

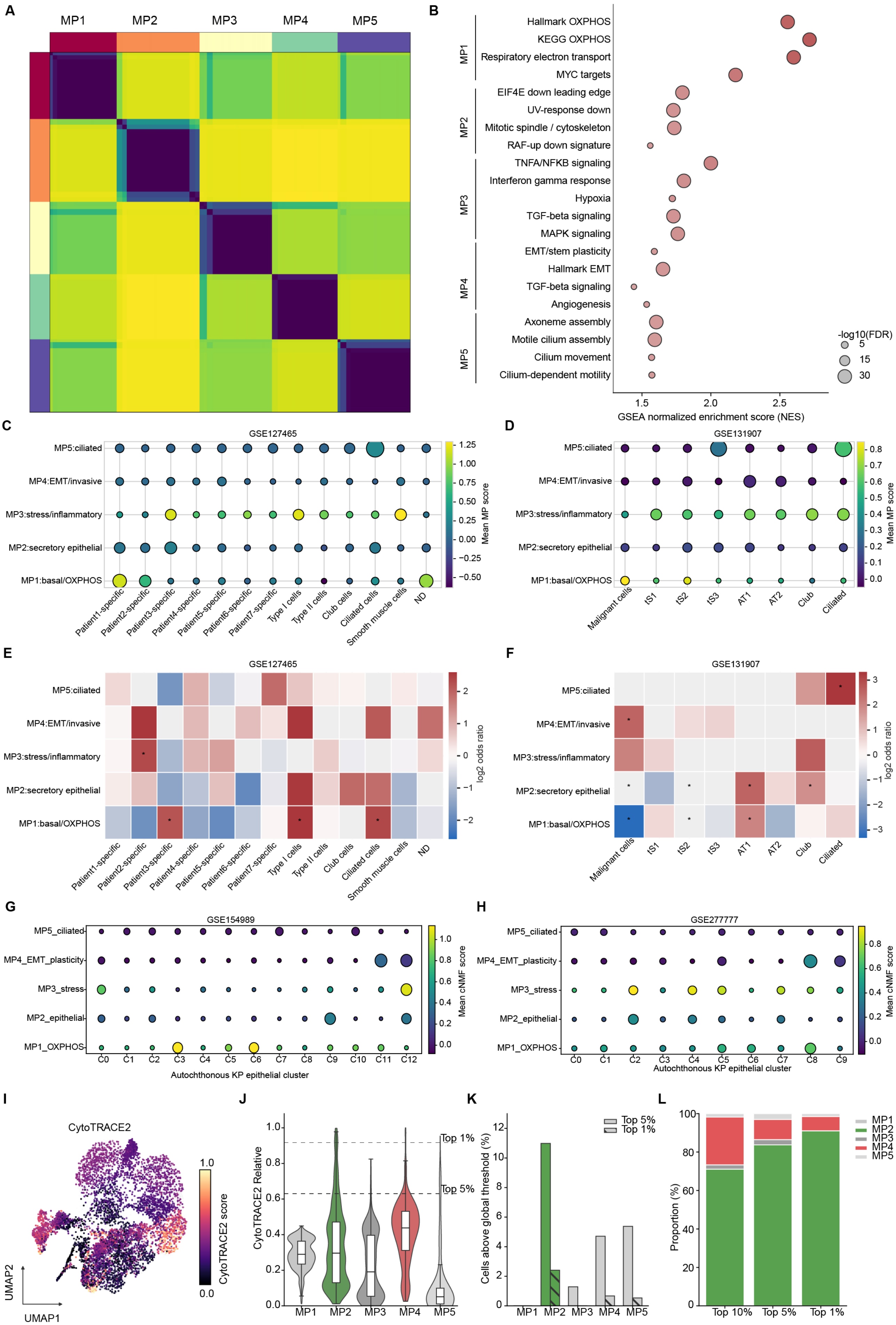

A
MP1
MP2
MP3
MP4
MP5
B
Hallmark OXPHOS
KEGG OXPHOS
Respiratory electron transport
MYC targets
EIF4E down leading edge
UV-response down
Mitotic spindle / cytoskeleton
RAF-up down signature
TNFA/NFKB signaling
Interferon gamma response
Hypoxia
TGF-beta signaling
MAPK signaling
EMT/stem plasticity
Hallmark EMT
TGF-beta signaling
Angiogenesis
Axoneme assembly
Motile cilium assembly
Cilium movement
Cilium-dependent motility
-log10(FDR)
5
15
30
1.5
2.0
2.5
GSEA normalized enrichment score (NES)
C
GSE127465
MP5:ciliated
MP4:EMT/invasive
MP3:stress/inflammatory
MP2:secretory epithelial
MP1:basal/OXPHOS
Patient1-specific
Patient2-specific
Patient3-specific
Patient4-specific
Patient5-specific
Patient6-specific
Patient7-specific
Type I cells
Type II cells
Club cells
Ciliated cells
Smooth muscle cells
ND
Mean MP score
D
GSE131907
Malignant cells
tS1
tS2
tS3
AT1
AT2
Club
Ciliated
Mean MP score
E
GSE127465
log2 odds ratio
F
GSE131907
log2 odds ratio
G
GSE154989
MP5_ciliated
MP4_EMT_plasticity
MP3_stress
MP2_epithelial
MP1_OXPHOS
C0 C1 C2 C3 C4 C5 C6 C7 C8 C9 C10 C11 C12
Autochthonous KP epithelial cluster
Mean cNMF score
H
GSE277777
C0 C1 C2 C3 C4 C5 C6 C7 C8 C9
Autochthonous KP epithelial cluster
Mean cNMF score
I
CytoTRACE2
1.0
0.0
CytoTRACE2 score
UMAP2
UMAP1
J
CytoTRACE2 Relative
Top 1%
Top 5%
MP1 MP2 MP3 MP4 MP5
K
Top 5%
Top 1%
Cells above global threshold (%)
MP1 MP2 MP3 MP4 MP5
L
Proportion (%)
Top 10%
Top 5%
Top 1%
MP1
MP2
MP3
MP4
MP5

Supplementary Figure 2. Meta-program annotation

A, cNMF program structure showing five reproducible meta-programs (MPs) across epithelial cells.

B, Gene-set enrichment analysis (GSEA) of the five epithelial MPs.

C, Projection of the five MP scores onto annotated epithelial and stromal cell states in GSE127465 [1].

D, Projection of the five MPs scores onto annotated epithelial cell states in GSE131907 [2].

E, Cell-type enrichment of metaprograms in GSE127465 [1], shown as log2 odds ratios. Asterisks mark significant enrichment.

F, Cell-type enrichment of metaprograms in GSE131907 [2], shown as log2 odds ratios. Asterisks mark significant enrichment.

G, Cell-type enrichment of metaprograms in GSE154989 [3], shown as log2 odds ratios. Asterisks mark significant enrichment.

H, Cell-type enrichment of metaprograms in GSE277777 [4], shown as log2 odds ratios. Asterisks mark significant enrichment.

I, UMAP projection of CytoTRACE2 scores in epithelial cells.

J, Distribution of relative CytoTRACE2 scores across dominant MP assignments.

K, Fraction of cells from each MP exceeding the top 5% and top 1% CytoTRACE2 thresholds.

L, MP composition of cells within the top 10%, top 5% and top 1% CytoTRACE2 score strata.

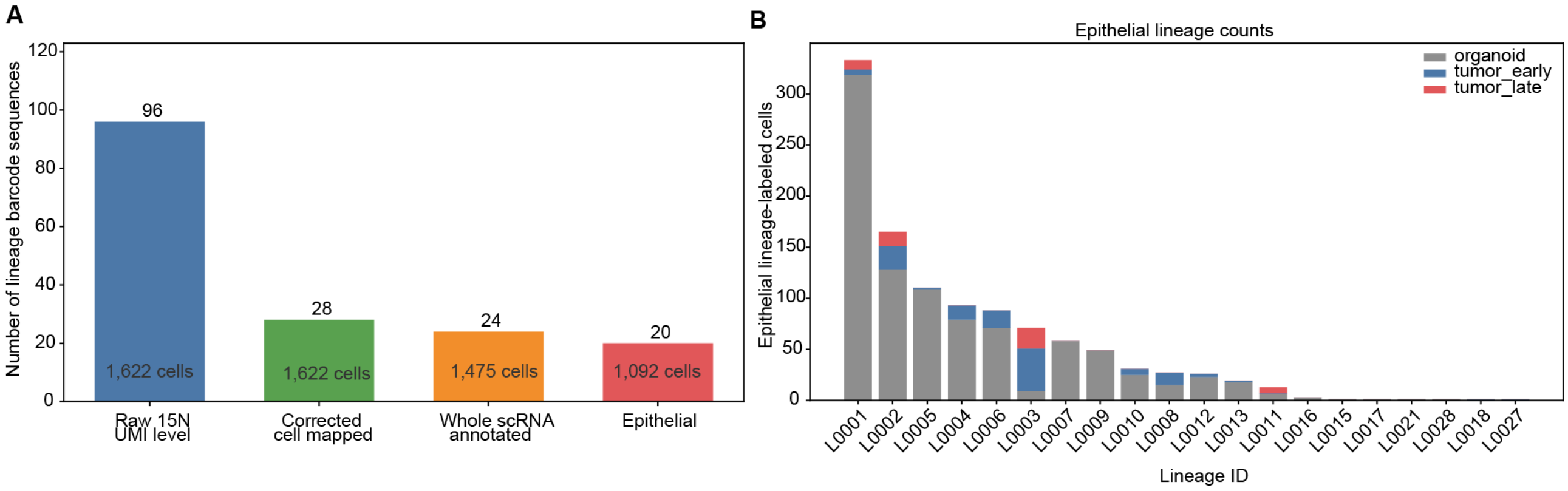

Supplementary Figure 3. Barcode recovery and epithelial lineage representation

A, Number of barcode sequences and associated cells retained after raw 15N UMI recovery, cell-barcode correction, whole-atlas annotation and epithelial-cell restriction.

B, Epithelial cell counts for each recovered lineage across organoid, early tumor and late tumor samples.

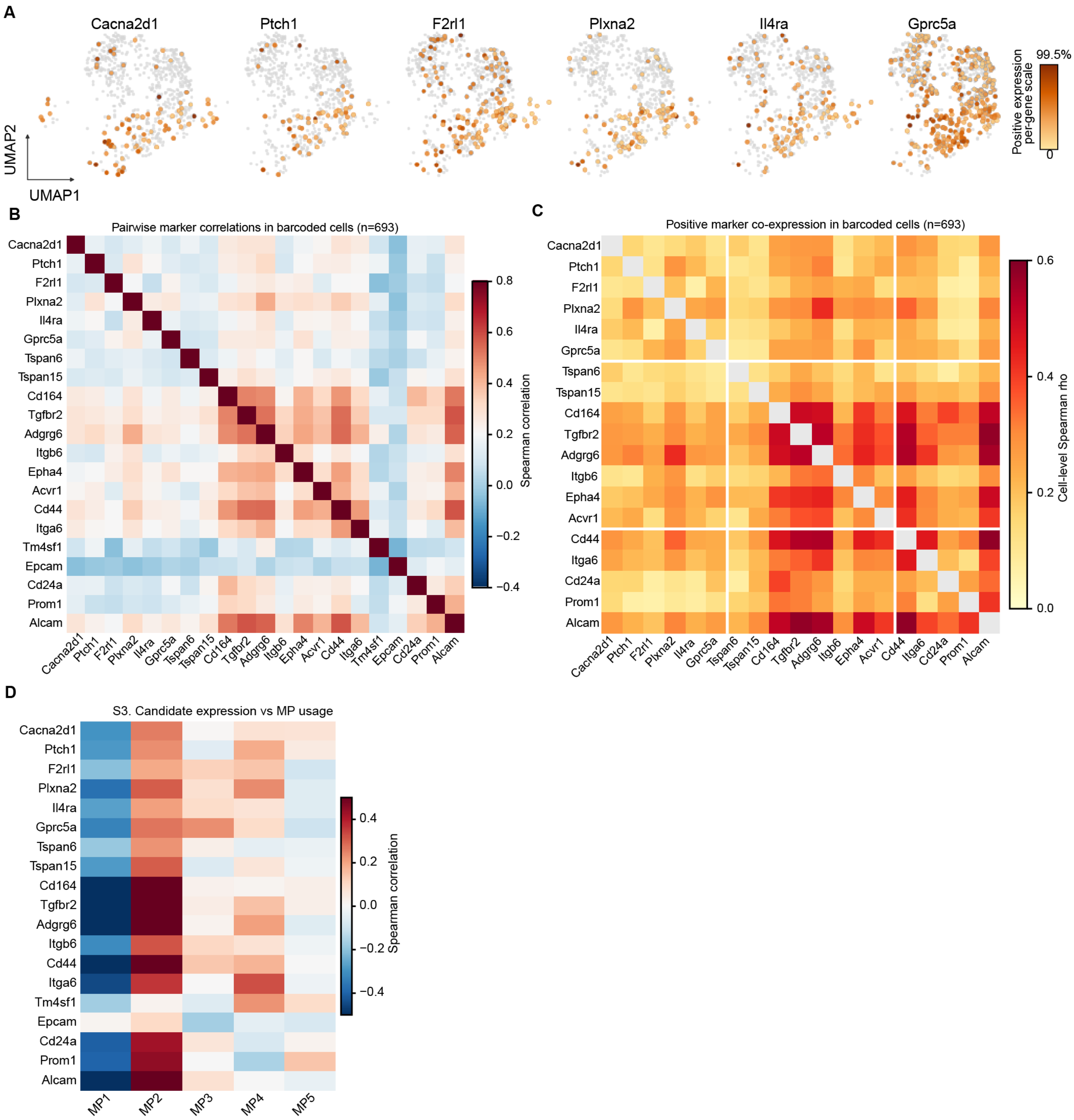

Supplementary Figure 4. Candidate surface markers show coordinated expression with the expansion-associated MP2/MP4

A, UMAPs showing expression distribution of selected candidate surface markers in barcoded baseline organoid cells.

B,C, Pairwise Spearman correlations among candidate marker expression levels in barcoded cells.

D, Spearman correlations between candidate marker expression and epithelial MP usage scores.

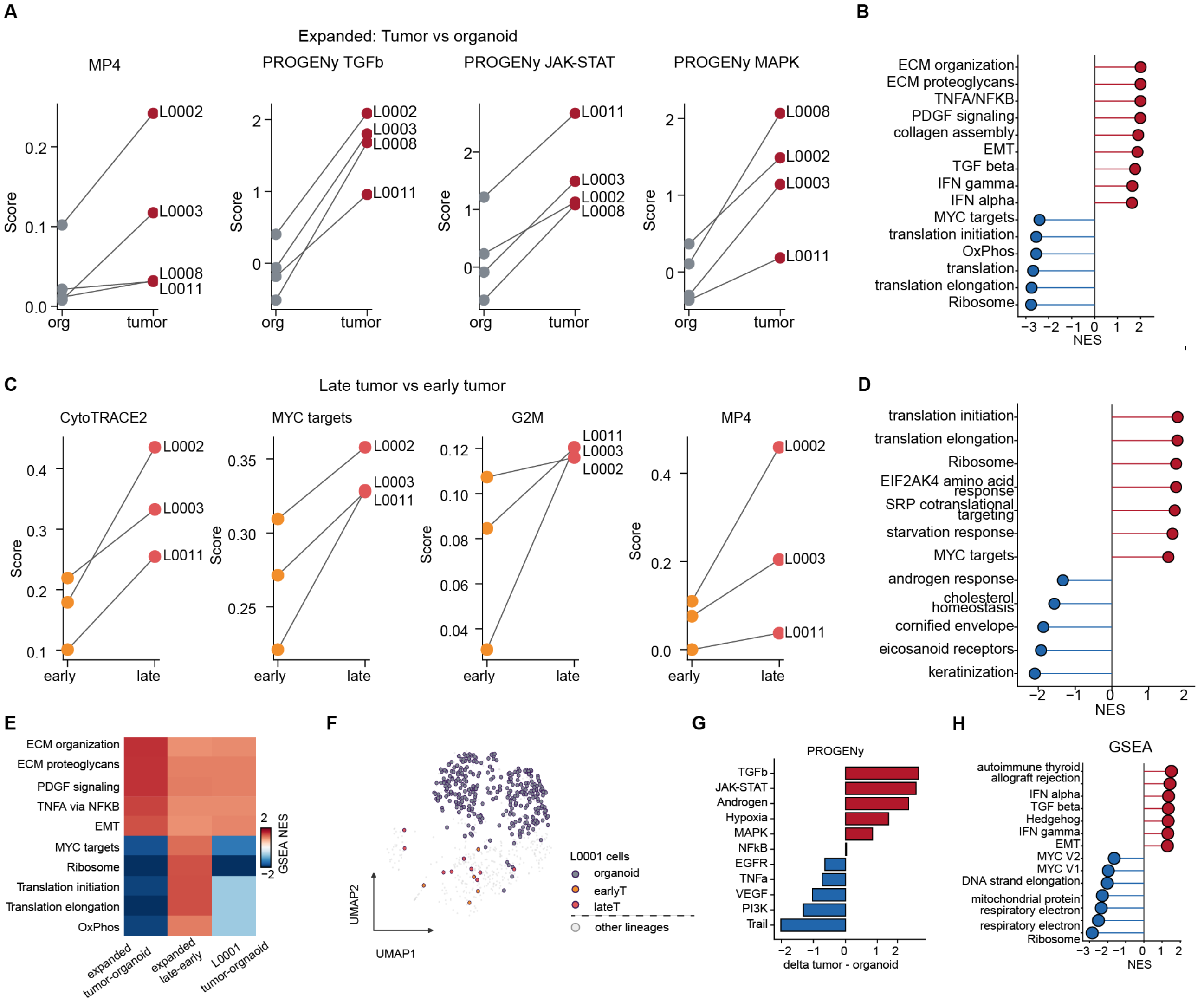

Supplementary Figure 5. Lineage-resolved transcriptional remodeling during tumor engraftment and progression

A, Paired lineage-level scores comparing expanded-lineage tumor cells with matched organoid cells for MP4 and PROGENy TGF-β, JAK-STAT and MAPK activity.

B, GSEA of expanded-lineage tumor cells versus matched organoid cells. Positive normalized enrichment scores indicate pathways increased in tumor cells.

C, Paired lineage-level scores comparing late tumor with early tumor cells for CytoTRACE2, MYC targets, G2M and MP4.

D, GSEA of late tumor versus early tumor cells within expanded lineages.

E, Heat map summarizing selected GSEA normalized enrichment scores across expanded tumor-versus-organoid, expanded late-versus-early and L0001 tumor-versus-organoid contrasts.

F, UMAP highlighting L0001 cells across organoid, early tumor and late tumor stages relative to other barcoded lineages.

G, PROGENy pathway activity changes in L0001 tumor cells compared with L0001 organoid cells.

H, GSEA of L0001 tumor cells versus L0001 organoid cells.

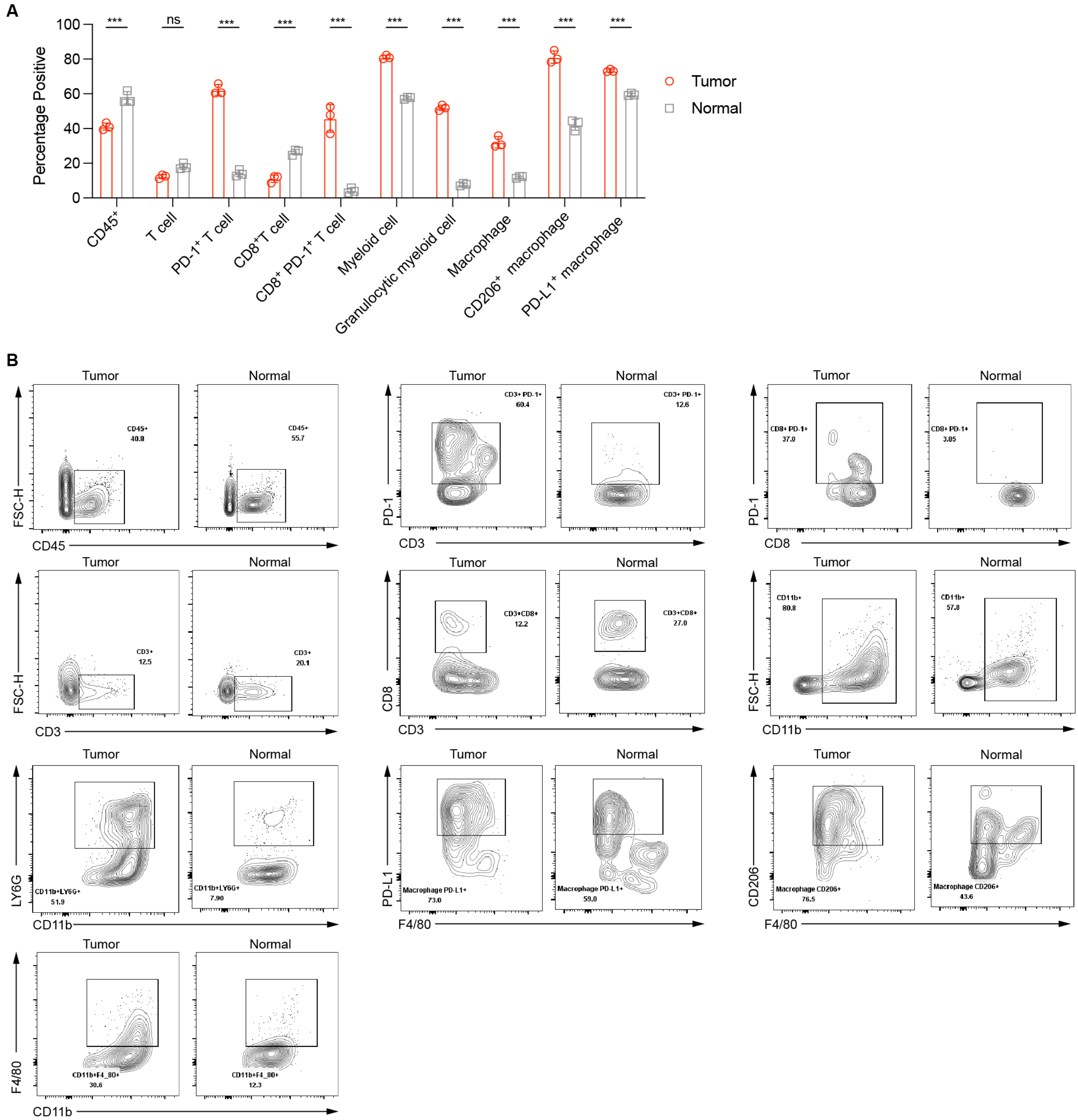

Supplementary Figure 6. Flow cytometry validates immune remodeling in p53K_LC_org tumors

A, Quantification of immune-cell populations in tumor (n=3) and matched normal lung samples (n=3) by flow cytometry. Bars show group summaries with individual samples overlaid. Data are mean ± SEM. Statistical comparisons were performed by one-sided Wilcoxon rank-sum test. ns, not significant; ***, $P < 0.001$.

B, Representative flow-cytometry gating plots for $CD45^+$ leukocytes, T cells, PD-1^+ T cells, $CD8^+$ T cells, $CD8^+$PD-1^+ T cells, $CD11b^+$ myeloid cells, $CD11b^+LY6G^+$ granulocytic myeloid cells, PD-$L1^+$ macrophages, $CD206^+$ macrophages and $CD11b^+F4/80^+$ macrophages in tumor and normal lung samples.

References

1 Zilionis, R. et al. Single-cell transcriptomics of human and mouse lung cancers reveals conserved myeloid populations across individuals and species. Immunity 50, 1317–1334. e1310 (2019).

2 Kim, N. et al. Single-cell RNA sequencing demonstrates the molecular and cellular reprogramming of metastatic lung adenocarcinoma. Nature communications 11, 2285 (2020).

3 Marjanovic, N. D. et al. Emergence of a High-Plasticity Cell State during Lung Cancer Evolution. Cancer Cell 38, 229–246.e213 (2020).

4 Chan, J. E. et al. Critical role for a high-plasticity cell state in lung cancer. Nature 651, 231–241 (2026).
